# Acteoside ameliorates hepatic ischemia-reperfusion injury *via* reversing the senescent fate of liver sinusoidal endothelial cells and restoring compromised sinusoidal networks

**DOI:** 10.7150/ijbs.87332

**Published:** 2023-09-18

**Authors:** Kexin Jia, Yinhao Zhang, Ranyi Luo, Runping Liu, Yajing Li, Jianzhi Wu, Kaihong Xie, Jia Liu, Shuo Li, Fei Zhou, Xiaojiaoyang Li

**Affiliations:** 1 School of Life Sciences, Beijing University of Chinese Medicine, Beijing, 100029, China.; 2 School of Chinese Materia Medica, Beijing University of Chinese Medicine, Beijing, 100029, China.

**Keywords:** hepatic ischemia-reperfusion injury, liver sinusoidal endothelial cell, senescence-associated secretory phenotype, acteoside, high mobility group protein 1.

## Abstract

Hepatic ischemia-reperfusion injury (HIRI), a common two-phase intersocietal reaction in liver surgery, typically leading to sustained liver dysfunction. During this process, liver sinusoidal endothelial cells (LSECs) are vulnerable to damage and exert senescence-associated secretory phenotype (SASP). However, how these SASP-LSECs secreted damage-associated molecular patterns (DAMPs) to impact the whole HIRI microenvironment and whether it can be reversed by therapeutics remains unknown. Here, we found that either HIRI surgery or hypoxia and reoxygenation (HR) stimulation forced LSECs into SASP and expressed HMGB1-dominated DAMPs, which were dramatically improved by acteoside (ACT). Additionally, hypoxic hepatocytes released excessive HMGB1 to LSECs and synergistically aggravated their SASP state. Mechanistically, HMGB1 bound with TLR3/TLR4 on LSECs, promoted the nuclear translocation of IRF1 and subsequent transcription of *cxcl1* and* Hmgb1*, leading to the chemotaxis of neutrophils and accelerating immune damage in a vicious circle. Notably, ACT or HMGB1 siRNA effectively disrupted HMGB1-TLR3/4 interaction, leading to IRF1 inhibition and repairing LSEC functions, which was largely reversed by HMGB1 stimulation and IRF1-overexpressed liposomes with LSECs-targeted hyaluronic acid-derivative conjugated in mice. Collectively, ACT reversed the senescent fate of LSECs and restored sinusoidal networks by targeting HMGB1-TLR3/4-IRF1 signaling, thus providing protection against HIRI and offering the potential for new therapeutics development.

## Introduction

Although liver transplantation has already become the ineludible treatment for end-stage liver disease and hepatic malignancies, it usually generates postoperative problems and remains an intractable clinical problem with the inevitable hepatic ischemia-reperfusion injury (HIRI). In simple terms, HIRI usually results from the reduplicative interrupted blood supply and restoration of perfusion and reoxygenation during liver surgery 1, 2. Furthermore, excessive oxidative stress, aberrant activation and recruitment of macrophages and neutrophils, pro-inflammatory reaction, and cellular apoptosis all participate in the pathogenesis of HIRI 3. Mechanistically, the persistent states of ischemia and anoxia aggravate the intercellular homeostasis destruction and decrease intracellular ATP levels, which contribute to the release of damage-associated molecular patterns (DAMPs) and destroy intrinsic cellular features and cell functions, especially for liver sinusoidal endothelial cells (LSECs) and hepatocytes 4. On the other hand, during the subsequent reperfusion phase, the gathered reactive oxygen species (ROS) broadly increases organelle damage in multiple hepatic cells, which further activates innate immune cells and causes an inflammatory cascade and ultimately intensifies the disorder of the hepatic sinusoid immune microenvironment 5. In light of the importance of preventing or alleviating HIRI in clinics, clarifying the underlying mechanisms and discovering effective strategies against HIRI and its induced multicellular damage are urgently needed.

In recent decades, the role of LSECs in the progression of HIRI has gained recognition. Since LSECs form a characteristic structural barrier between the liver sinusoids and hepatocytes, once injured, they will start the phenotypic transformation to the senescence-associated secretory phenotype (SASP), a type of persistent hypo-proliferative aging state with the injured discontinuous basement membrane, loss of open-fenestrae, increased cellular senescence markers and abnormal production of vasodilatory mediators like nitric oxide (NO) and several pro-inflammatory cytokines 6, 7. Furthermore, these senescent LSECs or called SASP-LSECs will deteriorate the hepatic sinusoid microenvironment and trigger the pathological intercellular communication with other hepatic cells including hepatocytes, contributing to the pathological processes in the two stages of HIRI 8, 9. Of note, high mobility group protein 1 (HMGB1)-dominated DAMPs released by damaged hepatocytes were reported to translocate from nuclei to the cytoplasm to trigger a potent inflammatory cascade in the HIRI process 10-12. In addition, LSECs not only sensed DAMPs or oxidative stress factors released by hepatocytes but also expressed multifarious adhesive molecules to accelerate immune damage during the HIRI process, which contributed to the worse phenotypic injury of LSECs and mediated aggravating pathological feedback in hepatic sinusoid 13-15. While the important role of injured LSECs in HIRI has been noticed, whether and how LSECs start the SASP transformation, impact the secretion of DAMPs and intercellular communication between LSECs and other hepatic cells during HIRI remain unknown.

Applicable therapies that avoid the cumbersome outcomings of HIRI remain limited, thus it is worth taking some inspiration from natural medicines. Acteoside (ACT), a phenylpropanoid glycoside extracted from natural dicotyledonous plants, has been considered to be promising in the treatment of acute liver injury, hepatitis, and cancer 16. Several studies stated that ACT may exert a direct protective effect on hepatocytes and improve steatosis and oxidative stress by inhibiting the nuclear factor kappa B pathway and further reducing the production of tumor necrosis factor-α (TNF-α) and ROS 17-21. Moreover, ACT inhibited the expression of cell adhesion molecules (CAMs), including intercellular CAM-1 (ICAM-1) and vascular CAM-1 (VCAM-1), to exert anti-inflammatory activities and protected the vascular endothelial cells from oxidative stress and free radicals 22-24. Additionally, it is worth noting that ACT inhibited the release of typical DAMPs and facilitated iNOS/NO production to lessen the inflammatory response 25. However, whether and how ACT exerts a noteworthy hepatoprotective function in repairing SASP-LSECs and multicellular communication in the hypoxic/ischemic hepatic microenvironment has not been studied.

Herein, by applying RNA sequencing of a single type of different hepatic cells and whole livers, molecular-scale interaction verification, and liposomes nanoparticle targeting delivery, we investigated whether ACT reversed the cell fate of SASP-LSECs during HIRI and how ACT regulated the intercellular crosstalk between senescent LSECs and other cells in hepatic sinusoid to improve HIRI. By providing evidence for the previously unrecognized role of SASP-LSECs in HIRI, this study not only provides new insights into the correlation between senescent LSECs and HIRI but also offers a basis for the development of potential therapeutic strategies for clinical HIRI prevention.

## Materials and methods

### Materials

ACT (61276-17-3) was obtained from Yuanye Bio-Tech (Shanghai, China), the type I Collagen (C3867), lipopolysaccharide (LPS, L260), Poly (I:C) (P9582), glycyrrhizic acid (GA, PHL89217), Williams' Medium E with L-glutamine (W4125), and other supplemental reagents for cell culture were from Sigma (St. Louis, USA). Purified HMGB1 protein was purchased from MedChemExpress (New Jersey, USA) and purified interferon regulatory factor 1 (IRF1) protein was obtained from Origene (Maryland, USA). Information about used antibodies was listed in **Table S1**.

### Animal studies

Mice (male, 9-10 weeks, 25 ± 2 g) were housed in sterile cages under a 12 h light-12 h dark cycle at a sustaining room temperature (22 ± 2°C) and had access to food regularly and sterile water *ad libitum* for 1-week adaptation. Before the HIRI operation, mice were divided into six groups randomly (n = 6): 1) sham group; 2) HIRI group; 3) HIRI + ACT low dose group (25 mg/kg); 4) HIRI + ACT medium dose group (50 mg/kg); 5) HIRI + ACT high dose group (100 mg/kg); 6) HIRI + N-acetylcysteine (NAC) group (100 mg/kg). Groups 3 to 6 were either pre-treated with ACT at different dosages or NAC and groups 1 and 2 were orally gavaged with phosphate-buffered saline (PBS) for 7 days. At the 8^th^ day of administration, in strict accordance with the time-controlled PBS or ACT administration, mice in groups 2 to 6 were given gaseous anesthesia with isoflurane and then underwent the HIRI surgery. In brief, mice were cut through the abdomen and their liver cephalad lobes were interrupted by an atraumatic clip to induce hepatic ischemia with a kind of faded tawny color, and after 1 h, the clip was removed to initiate the liver reperfusion. In the HMGB1-stimulated *in vivo* experiments (**Fig. 8**), mice were divided into 4 groups (n = 6): 1) sham group; 2) HIRI group; 3) HIRI + HMGB1 group; 4) HIRI + HMGB1 + ACT group (50 mg/kg). Groups 2 and 3 were administrated with HMGB1 recombinant protein (100 μg/kg) by tail vein injection 2 h before HIRI operation. Mice in group 4 were pretreated with ACT as previously described. In the caudal injection of hyaluronic acid (HA)-coupled liposomes nanoparticle for delivering IRF1 overexpression plasmid to mice (**Fig. 9**), mice were divided into 4 groups (n = 6): 1) sham group; 2) HIRI group; 3) HIRI + liposome (Lipo)-IRF1 group; 4) HIRI + Lipo-IRF1 + ACT group (50 mg/kg). Briefly, the plasmid of mouse IRF1 was synthesized by Hanbio (Shanghai, China; Primers: 5'-GUGAAGGAUCAGAGUAGGA-3', 3'-GUGAAGGAUCAGAGUAG GA-5') and the IRF1 liposomes labeled with polyethylene glycol (PEG) was prepared by lipid film hydration. After IRF1 liposomes suspension were prepared, the liposomal-PEG suspension (100 μl) was added the HA derivative (0.1 mol % lipids) labeled with DIR iodide according to a reported method 26, then the suspension was incubated for 0.5 h at room temperature and the material from fractions trapped in the filter was resuspended with PBS to achieve the desired dose of the HA-Lipo-IRF1 (100 μl suspension in 10 ml PBS). Groups 3 and 4 were intravenously injected with HA-Lipo-IRF1 (100 μl/20g) at 2 h before HIRI operation. Mice in group 4 were pretreated with ACT as previously described. All mice were sacrificed after 6 h post-operation to harvest the serum and liver tissue for subsequent studies. All animal procedures were approved by the Ethics Committee of the Institutional Animal Care and Use Committee of Beijing University of Chinese Medicine.

### Cell isolation, culture, treatment and hypoxia and reoxygenation (HR) model establishment

According to our previous research, collagenase perfusion was used to separate primary hepatocytes, which were then cultivated in Williams' Medium E with dexamethasone and L-thyroxine 27. Additionally, mouse primary LSECs were obtained by percoll gradient separation twice from the residual cell suspension, and then been separated from Kupfer cells according to different cell adherent times in the same culture dish. Primary LSECs, the cell lines of BRL-3A (hepatocytes) and LSECs (BNBIO., Beijing, China) were cultured in Dulbecco's Modified Eagle Medium (DMEM) with the supplement of 10% FBS, 1% 100 U/mL penicillin G, and 100 μg/mL streptomycin. Generally, the HR model is an *in vitro* model to mimic the pathological injury of HIRI. To construct the HR model* in vitro*, LSECs were firstly incubated in a microaerophilic system (Heal Force, Shanghai, China) containing 94% N_2_ gas, 5% CO_2_ and 1% O_2_ for 16 h and then were cultured under 37 °C and 5% CO_2_ environment for 8 h to allow reoxygenation as previously reported 28. Furthermore, for the co-culture system of LSECs and hepatocytes under HR insult, briefly, after DMSO or ACT pretreating (25, 50 and 100 μM) for 8 h, BRL cells were placed in the sterile HR environment for 16 h and back to an oxygen-rich culture environment for 8 h. Later, CM from BRL cells in different groups was collected and then treated LSECs for another 12 h. In the agonist experiments, after DMSO or ACT (50 μM) pretreatment, LSECs were either given with Poly (I:C) (5 μg/ml), LPS (100 ng/ml), recombinant HMGB1 protein (100 ng/ml) and recombinant IRF1 protein (100 ng/ml) for 8 h. The primary neutrophils were separated from mouse bone marrow by applying a commercial neutrophil extraction kit (Solarbio, Catlog: P8550, Beijing, China). The primary neutrophils were cultured for about 4 h, then incubated with LPS (50 ng/mL) and/or HR-LSEC CM, with ACT treatment (25, 50 and 100 μM) for another 8 h. At the end of different cultures and treatments, conditioned medium or cells were collected respectively for ELISA, immunofluorescence experiments and qPCR detections.

### RNA-Sequencing of mice liver, hepatocytes and LSECs

The total RNA from mouse livers in different groups was isolated, purified and quantitated by NanoRhatometer^@^ spectrophotometer (IMPLEN, USA). Then the RNA integrity was measured by Nano Assay (Agilent, Shanghai, China), and the purification of total RNA was performed by magnetic bead under the incubation of poly-T oligo. Then the AMPure XP Agencourt (Beckman Coulter Life Science, USA) was applied cDNA after synthesis for the purification and enrichment of the cDNA library fragment. Then RNA-sequencing library of mouse liver was established with the help of Illumina Novaseq (Illumina, California, USA) 29. For cells RNA-sequencing, primary murine hepatocytes and LSECs were isolated as described above. The total RNA of hepatocytes and LSECs were isolated by applying Trizol classical extraction method, quantified by NanoRhatometer spectrophotometer (IMPLEN, USA) and reverse-transcribed to cDNA by applying the cDNA reverse transcription kit from Vazyme company (Nanjing, China). Furthermore, the cells-RNA sequencing library was established and operated on the Illumina Novaseq platform as same as described total liver procedures. Subsequently, all involved samples for RNA-sequencing were normalized and differentially expressed genes (DEGs) were analyzed by applying the edgeR software. Gene ontology (GO) enrichment analysis of DEGs was operated by cluster Profiler R package and signaling pathway analysis was analyzed by STRING (https://string-db.org/) and kyoto encyclopedia of genes and genomes (KEGG) database (https://www.kegg.jp/). For weighted gene co-expression network (WCGNA) analysis, the WGCNA arithmetic was applied to evaluate the hot coding genes and top modules. Then clustering the samples from different groups and calculating the distance of chosen genes after removing the redundancy genes. The R package was applied to establish the final network, and Bioinformatics (https://www.bioinformatics.com.cn/) was used for generating the cluster heatmap, violin plots and circle graph. Benjamini & Hochberg adjusted *p* value < 0.05 was used to present a significant difference.

### Fluorescein isothiocyanate-labeled formaldehyde-treated serum albumin (FITC-FSA) uptake assay

The FITC-FSA solution (10 mg/ml) was prepared according to previous description 30. In short, FSA was melted in PBS for 3 days and further labeled with FITC to get the FITC-FSA solution. For the intravital FITC-FSA uptake assay, the FITC-FSA solution was diluted by 1× PBS to 1 mg/ml and injected into the mice with 100 µl volume. After 1h injection, mouse livers were rapidly harvested on ice and frozen-embedded in OCT-freeze medium in the pre-cooling freezing microtome under the dark. Next, the liver sections were sliced as soon as possible and were incubated with DAPI and observed immediately under a 488-nm laser. For cellular staining, LSECs were incubated with 100 µg/ml FITC-FSA after ACT pretreatment and HR stimulation. Then, cells in different groups were also fixed in 4 % formaldehyde and DAPI, and examined by confocal fluorescence microscopy (Olympus FV3000, Tokyo, Japan).

### HMGB1-toll like receptor 3 (TLR 3)/4 interaction and protein binding analysis

To explore the interaction between HMGB1 and TLR3 and TLR4, the RCSB PDB database (https://www.rcsb.org/) was performed for identifying existing or/and potentially associated interaction. Then the PyMOL software (https://pymol.org/2/) and the Auto- DockTools (https://en.freedownloadmanager.org/Mac-OS/AutoDockTools-FREE.html) were utilized for visualizing and modifying the protein structure. The docking simulations were excused by processed protein structures of the ligand and the target proteins were analyzing docked were conducted by Molecular Operating Environment docking system. The PyMOL was utilized to visualize the proteins docking complexes.

### Electrophoretic mobility shift assay (EMSA) experiment

The double-stranded chemokine (C-X-C motif) ligand 1 (CXCL1)-targeted IRF1 probes were predicted and designed by ENSENMBL Genomes (http://asia.ensembl.org/index.html) and JASPER database (https://jaspar.genereg.net/), then the probes were end-labeled with biotin from Beyotime Biotechnology (Shanghai, China). The binding reactions were reacted in a prepared mixture containing purified IRF1 proteins and 5 × gel shift binding buffer from Beyotime. For competition reactions, unlabeled CXCL1 or Mut CXCL1 probes were added to the above mixture at a concentration 100-fold higher than that of the labeled probe at room temperature for 20 min incubation. For the supershift sample, IRF1 antibody was added to the relative mixture and incubated for another 10 min. Subsequently, the pre-labeled probe was added to relative mixture and incubated for 0.5 h. All products were then loaded by electrophoresis in a nondenaturing polyacrylamide gel prepared in Tris-borate-EDTA. Last, the obtained gel was dried for autoradiography.

### Drug affinity-responsive target stabilization assay (DARTS) assay

For the DARTS assay, approximately 1 × 10^6^ untreated LSECs were first lysed on ice for 0.5 h. The concentration of ACT (50 μM) was supplemented into the prepared cell lysate with 500 μg total protein. Then, cellular protein samples were separately mixed followed by 2-hour incubation for adequately binding the target of protein-ligand. Different lysate samples were further digested by pronase (Yuanye Bio-Tech, Shanghai, China) at the ratio of 1 : 300, 1 : 1000, 1 : 2000, 1 : 3000 and 1 : 10000 for precisely 30 min and reacted with the loading buffer for subsequent Western blot analysis.

### Cellular thermal shift assay (CETSA) experiment

For the CETSA experiments involved in **Fig. 6**, LSECs were lysed by repeated freeze-thaw method and further separated for two parts: one part was incubated with the reaction solvent as negative control and the second part was treated with ACT (50 μM) for 0.5 h as the ACT-treated group. After relative incubation, these two lysates were heated at sequentially rising temperatures (ranging from 37°C to 70°C). After heating, the supernatants in lysates were collected and used to analyze the TLR3 and TLR4 abundance level by Western blot assay.

### Statistical analysis

All experimental data are shown as the mean ± SD and repeated for at least three times. The data comparison was completed by a one-way ANOVA and the Tukey's post-hoc test. Data analysis for all figures was completed using GraphPad Prism 8.1 Software (La Jolla, CA, USA). *P* < 0.05 was used to characterize significant differences.

Additional information for involved methods and other details were provided in the **Supplementary file**.

## Results

### ACT obviously restored the hepatic sinusoidal structure in HIRI mice

**Figure 1:**
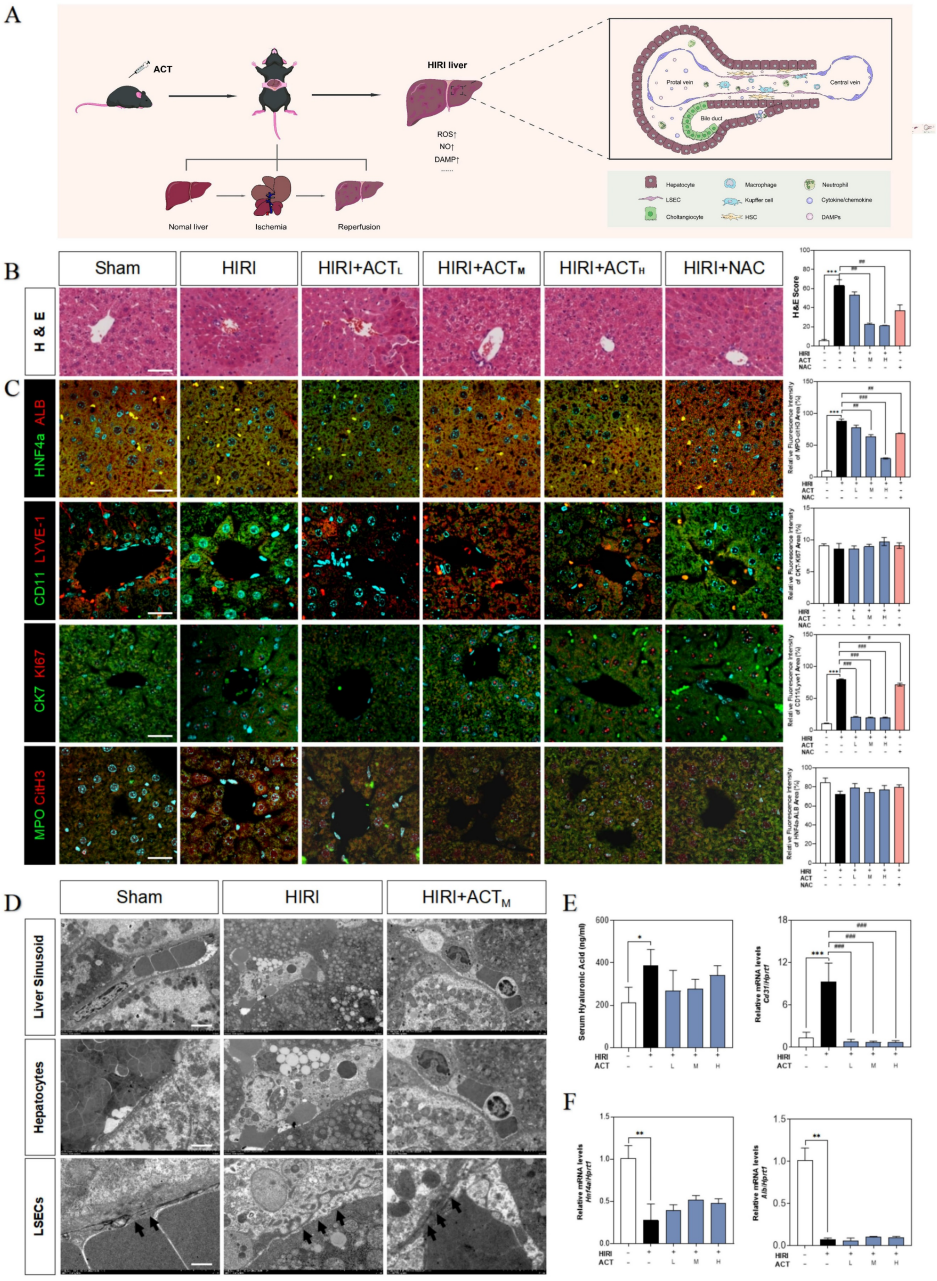
**ACT alleviated the hepatic sinusoidal microenvironment in the HIRI mice.** (**A**) Flow chart of animal experiment and fundamental structure of hepatic sinusoid. Representative images of (**B**) H & E staining, (**C**) immunofluorescence staining for HNF4α and ALB, CD11 and LYVE-1, CK7 and Ki67, MPO and citH3 in the mouse liver, scale bar = 40 μm. (**D**) Representative images of TEM, scale bar = 5 nm. (**E**,** left panel**) Serum ELISA results of HA in mice. Relative mRNA levels of *cd31* (**E**,** right panel**), (**F**) *Hnf4a* and *Alb* were measured by qPCR and further normalized with *hprt1*. Statistical significance: *P < 0.05, **P < 0.01, ***P < 0.001, compared with the sham group; ^#^*P* < 0.05, ^##^*P* < 0.01, ^###^*P* < 0.001 compared with the HIRI group (n = 6).

Initially, we evaluated the therapeutic effects of ACT on HIRI and preliminarily characterized the more vulnerable cell populations in injured livers (**Fig. 1A**). Hematoxylin and eosin (H & E) staining (**Fig. 1B**), serum transaminases (increased aspartate aminotransferase [AST], alanine aminotransferase [ALT], lactate dehydrogenase [LDH] and decreased NO) and liver function indicators (increased malonaldehyde [MDA], decreased superoxide dismutase [SOD] and hepatic NO) detection (**Fig. S1A** and** 1B**) showed the architectural and functional liver damage during HIRI progression. Notably, compared with the HIRI group, different dosages of ACT (25, 50 and 100 mg/kg) markedly ameliorated the histopathological injury including characteristic sinusoidal congestion, vacuolization, hepatocytes necrosis, and evident oxidative stress, which were similar to or even better than NAC administration (100 mg/kg). According to the noticeable structural damage of liver sinusoidal region, we further characterized the dominating cell types impacted by HIRI (transmission electron microscope [TEM] examination for whole liver, immunofluorescent staining like albumin [ALB] and hepatocyte nuclear factor 4 [HNF4α] for hepatocytes, lymphatic vessel endothelial hyaluronic acid receptor 1 [LYVE-1] and CD11 for LSECs and macrophages, keratin 7 [CK7] and antigen identified by monoclonal antibody Ki 67 (KI67) for cholangiocytes, myeloperoxidase [MPO] and citrullinated H3 [CitH3] for neutrophils) and the intervening pattern of ACT. As shown in **Fig. 1C**, phenotypic changes, and intrahepatic damage mainly occurred in LSECs that were located in the liver sinusoid and surrounded by inflammatory neutrophils during the HIRI, which were markedly remitted by ACT pretreated. Furthermore, TEM staining was applied to evaluate the hepatic ultrastructure change under HIRI for verifying the main injured cell types (**Fig. 1D**). As excepted, the hepatic sinusoidal space of HIRI mice was extruding and destructive: hepatocytes were visibly swollen with tanglesome ultrastructure and inflammatory cells gathered, and particularly, LSECs loosed its characteristic phenotype of fenestration, while both damaged LSECs and hepatocytes could be well-preserved by the premedication with ACT (50 mg/kg). Meanwhile, ACT administration dramatically restrained the hyaluronic acid (HA) secretion and the platelet and endothelial cell adhesion molecule 1 (PECAM1/CD31) levels compared with the HIRI group, suggesting a protective effect of ACT on the damaged physiological function of LSECs (**Fig. 1E**). Similarly, ACT exerted a hepatocellular-protective effect through slightly upturning the expression of hepatocytes makers *hnf4a* and *alb* and inhibited the pathologic LSECs functional markers of hypoxia-inducible factor 1 subunit alpha (*hif1a*), Krüppel-like transcription factor 2 (*klf2*) and *cd31* (**Fig. 1F** and **Fig. S1C**). Taken together, the changes of microstructure in hepatic sinusoid and makers of hepatic cell subsets indicated the vital role of damaged LSECs and hepatocytes played in HIRI as well as the noticeable importance of ACT in repairing the injured hepatic sinusoidal microenvironment.

### ACT primarily affected the fate of LSECs and hepatocytes in HIRI

**Figure 2:**
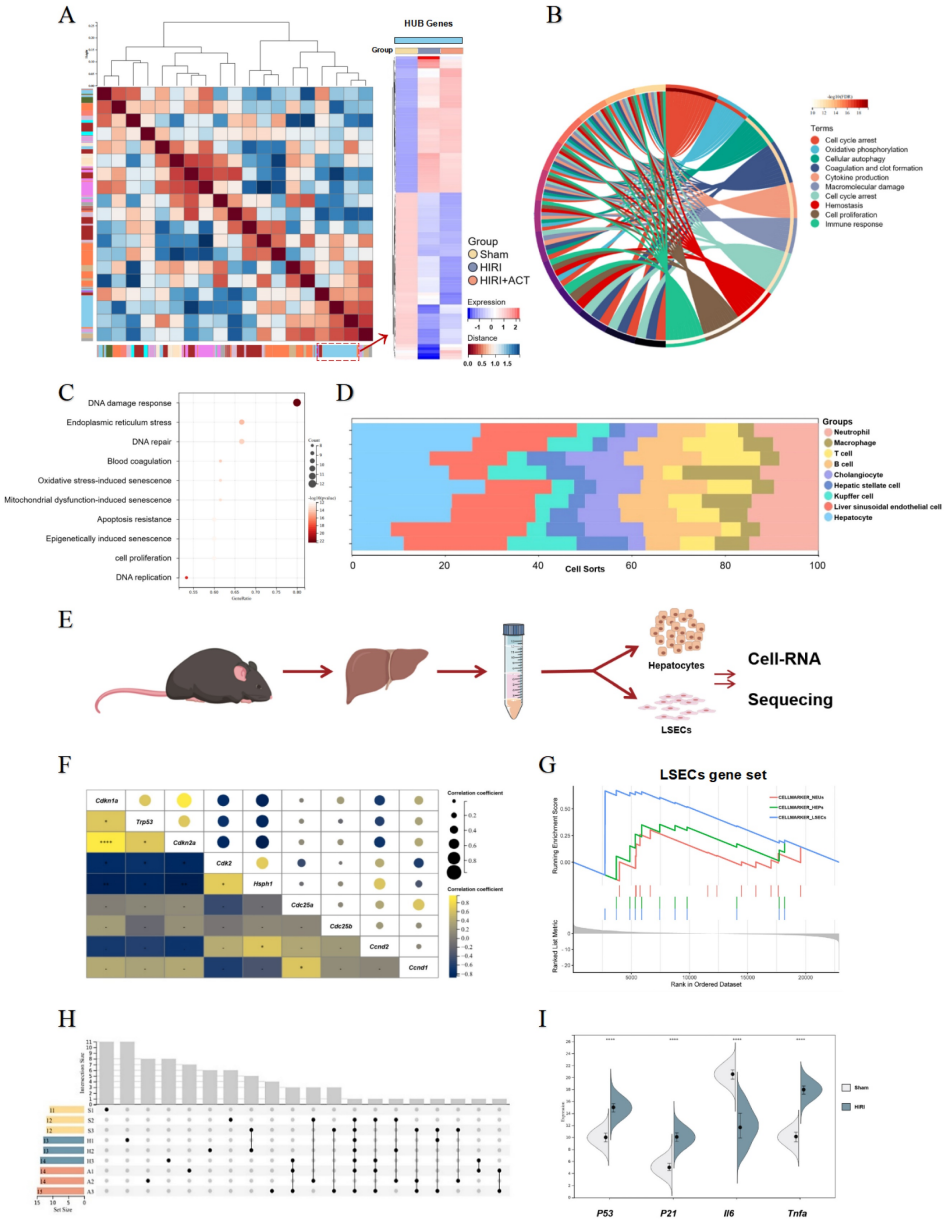
** The RNA-sequencing analysis of mouse liver, primary hepatocytes and primary LSECs.** (**A**) The gene heatmap of RNA-sequencing analysis of mice liver. (**B**) The clusters of liver ischemia and reperfusion-related molecular function. (**C**) The bubble diagram of liver ischemia and reperfusion-related biological process. (**D**) The dominant cells analysis in mice liver. (**E**) Representative flow chart of isolated hepatocytes and LSECs for cellular mRNA sequencing. (**F**) The top gene-expression of SASP genes. (**G**) Gene set of dominant cells analysis with SASP genes. (**H**) The relative expression of SASP-related genes in different groups. (**I**) The genes expression of *p53*, *p21*, *iI6*, *tnfa* in different groups.

For further defining the underlying pathomechanism of HIRI and the protective roles of ACT, the transcriptome sequencing in the overall level of mouse whole livers and in the microscopic level of hepatic-cell subsets (mainly for primary isolated hepatocytes and LSECs) were processed respectively and described in **'Method'** part. Specifically speaking, at the overall level, we identified enriched transcripts from mouse livers using the non-parametric Wilcoxon rank-sum test (**Fig. 2A**).

The top DEGs in liver from different group mice were shown and approximately 802 genes were significantly changed in HIRI liver compared to sham mice (**Fig. S2A**). Furthermore, we performed GO and KEGG analyses to identify the dominating biological process (**Fig. 2B** and **Fig. S2B**) and surprisingly, found the enrichment of immunologic reaction genes and SASP-related genes (oxidative stress-induced senescence, mitochondrial dysfunction-associated senescence, epigenetically induced senescence, cell proliferation) in the HIRI transcriptomic data (**Fig. 2B** and** 2C**). Meanwhile, these significant altered genes were mainly enriched in hepatocytes and LSECs in another cell hallmarked cluster analyses (**Fig. 2D**), which was consistent with the injured histopathology manifestation in whole livers. Subsequently, we started in-depth RNA analysis of the isolated primary hepatocytes and LSECs (**Fig. 2E**) and attempted to figure out which cell subsets in the whole liver were correlated with the SASP-related genes more specific and affiliated. Altogether, the expression of tumor protein p53 (*P53*), cyclin dependent kinase inhibitor 1A (*P21*), interleukin 6 (*Il6*) and *Tnfa* were significantly enriched in senescence-related pathways (**Fig. 2F** and **Fig. S2C**), and cell-hallmark analysis demonstrated that the cluster of the LSECs gene set was dominating in HIRI liver mice (**Fig. 2G**), which offered a potential cellulate target for anti-aging of ACT. Performing overrepresentation analysis and gene set enrichment analysis (GSEA) with established gene sets for cell clusters and SASP genes, we systematically confirmed that LSECs showed a tighter relevance with SASP trend (*p53*, *p21*, *il6* and *tnfa*) (**Fig. 2H** and **2I**) 31. These results indicated that SASP-related genes were up-regulated in the whole HIRI liver especially the LSECs rather than hepatocytes, but hepatocytes still underwent a persistent injured pattern, indicating that hepatocytes might play an essential role in regulating the function of SASP-LESCs.

### ACT reversed the senescent phenotype of LSECs under HIRI insult

**Figure 3:**
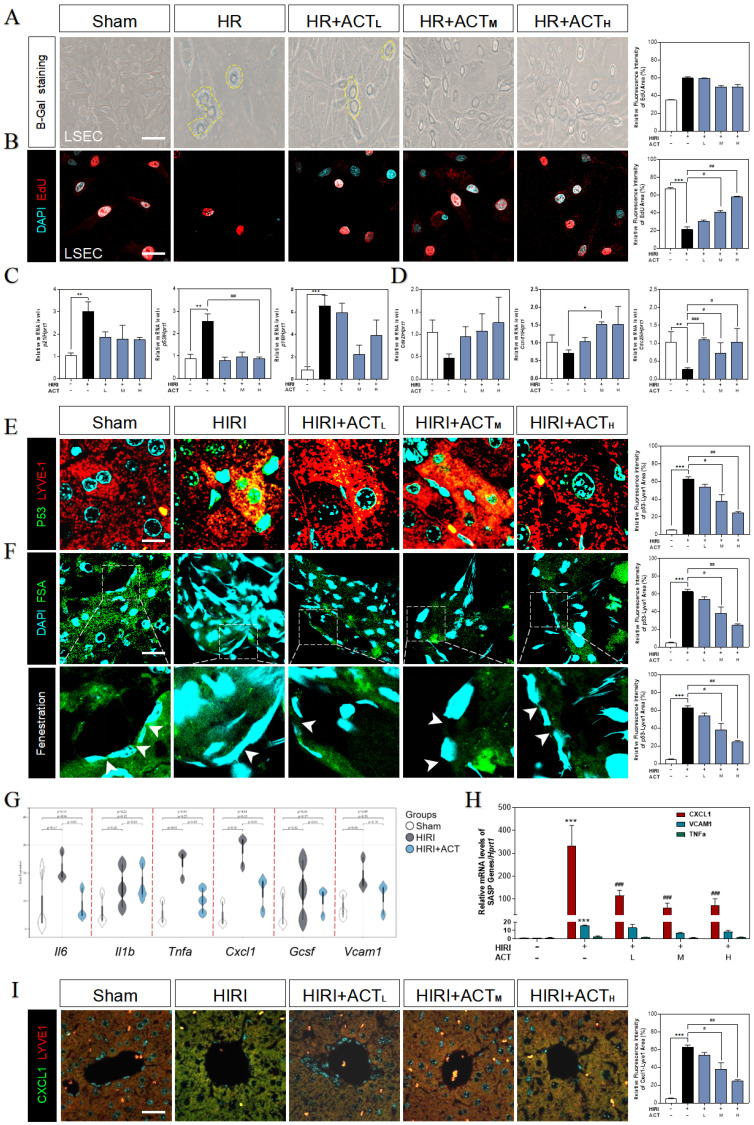
** ACT reversed the senescent state of LSECs**. (**A**) Representative images of β-Gal^+^ staining and (**B**) EdU staining of LSECs (Scale bar = 100 μm). Relative mRNA levels of (**C**) *p21*, *p53*, *p16*, (**D**)* cdk2*, *ccnd1*, *cdc25*, (**H**) *cxcl1*, *vcam1* and *Tnfa* were measured by qPCR and further normalized with *hprt1* in mouse livers. Representative images of immunofluorescence staining for (**E**) P53 and LYVE-1, (**F**) FITC-FSA staining and (**I**) CXCL1 and LYVE-1 of mouse liver (Scale bar = 40 μm). (**G**) The gene expression of SASP-related genes (*il6*,* il1b*, *tnfa*, *cxcl1*, *vcam1*) in the RNA-sequencing analysis results of mouse liver. Statistical significance: *P < 0.05, **P < 0.01, ***P < 0.001, compared with the sham group; ^#^*P* < 0.05, ^##^*P* < 0.01, ^###^*P* < 0.001 compared with the HIRI group (n = 6).

Due to the highly positive relation between aged LSECs and HIRI supported by sequencing data from mouse liver, the senescent status in HR and ACT-treated LSECs was then tested in different aspects. We found an obvious upregulation of SA-β-Gal^+^ cells in HR-insulted LSECs (**Fig. 3A**) and observed the cells quiescence in LSECs *via* negative incorporation of 5-ethynyl-2'-deoxyuridine (EdU) (**Fig. 3B**), which were inhibited by ACT administration and interestingly not noticed in hepatocytes under same situation (**Fig. S3A** and** S3B**). Besides, we detected relative critical markers of cell senescence or cell cycle and showed the upregulated mRNA levels of* p21*, *p53* and cyclin dependent kinase inhibitor 2A (*p16*) but downregulated levels of cyclin-dependent kinase 2 (*cdk2*), cell division control protein 25 (*cdc25*) and cyclin D1 (*ccnd1*) in livers after HIRI insult, which were largely corrected by ACT (**Fig. 3C** and** 3D**). We further wanted to confirm our RNA sequencing results of a single type of cells and explored whether the senescent trend was mainly occurred on LSECs instead of hepatocytes. The co-stained immunofluorescence results of senescence maker P53 with LYVE-1 (an LSEC maker) showed a strong correlation of senescence with the LSECs in HIRI mice liver but not in ACT-treated mice (**Fig. 3E**). Simultaneously, the scavenger receptor-mediated FITC-FSA uptake assay was applying for illustrating the endocytosis of LSECs. Compared with the remarkable fenestration and endocytosis showed in the sham group, FITC-FSA was un-efficiently uptaken by the damaged LSECs in HIRI group whereas the uptake of FSA was visibly improved in all HIRI + ACT groups (**Fig. 3F**). Moreover, some pro-inflammatory SASP genes responsible for responding senescent cells were also noticed in sequencing data. *Cxcl1* showed the highly ascending trend rather than other inflammatory factors like *il6*, *vcam1* and* tnfa*, which could be downregulated by ACT (**Fig. 3G** and **Fig. S3C**) and similarly, were verified by the qPCR detection (**Fig. 3H**). Furthermore, the immunofluorescence staining of CXCL1, and LYVE-1 showed an observable relevance after HIRI operation, and ACT significantly downregulated the expression of CXCL1 in a dose-dependent way (**Fig. 3I**). These data indicated that ACT could not only reverse the senescent trend of LSECs but also inhibit the release of CXCL1-dominant inflammatory SASP, which also remind us to notice the follow-up immune situation in the sinusoidal microenvironment.

### ACT prevented the trend of SASP-LSECs induced by hepatocyte-releasing HMGB1

**Figure 4:**
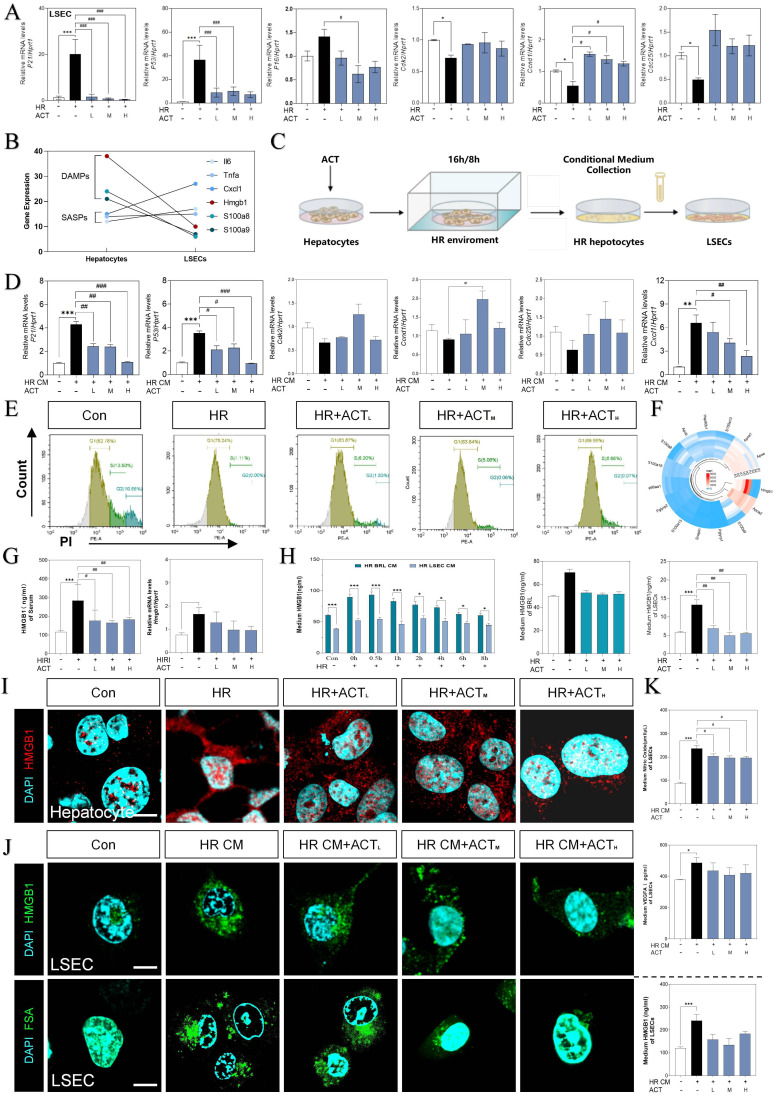
** HMGB1-oriented DAMPs secreted from hepatocytes rather than LSECs.** (**A**) Relative expression of mRNA levels of *p21*, *p53*,* p16*,* cdk2*, *ccnd1* and* cdc25* were measured by qPCR and further normalized with *hprt1* in HR and ACT-treated LSECs. (**B**) Gene expression of DAMPs (*hmgb1*, *s100a8*, *s100a9*) and SASPs (*il6*,* tnfa*, *cxcl1*) in primary hepatocytes and LSECs. (**C**) The co-culture flow chart of hepatocytes and LSECs under HR environment with ACT treatment. (**D**) Relative expression of mRNA levels of *p21*, *p53*,* cdk2*, *ccnd1*, *cdc25* and *cxcl1* were measured by qPCR and further normalized with *hprt1* in HR CM-treated LSECs. (**E**) Flow cytometry results of AV/PI of LSECs under HR CM insult and ACT treatment. (**F**) Ring Charts of dominant DAMPs gene analysis. (**G**) ELISA results of HMGB1 in mouse serum and relative mRNA expression of *hmgb1* of mouse liver normalized with *hprt1*. (**H**) ELISA results of HMGB1 in HR medium of hepatocytes and LSECs at different time points and ELISA results of HMGB1 in HR medium of hepatocytes and LSECs with ACT treatment. Representative images of immunofluorescence staining for HMGB1 of (**I**) hepatocytes and (**J**,** upper panel**) LSECs, and (**J**,** lower panel**) FITC-FSA of LSECs, scale bar = 100 μm. (**K**) ELISA results of NO and VEGFA in the CM of LSECs under the treatment of CM from the HR hepatocytes and ACT. Statistical significance: *P < 0.05, **P < 0.01, ***P < 0.001, compared with the control group; ^#^*P* < 0.05, ^##^*P* < 0.01, ^###^*P* < 0.001 compared with the HIRI group (n = 6).

For more directly illustrating that the cellular senescence occurred on LSECs far more than hepatocytes under the HIRI insult, qPCR assay was applied to detect the expression of *p21*, *p53*,* p16*, *cdk2*, *ccnd1* and *cdc25* both in LSECs and hepatocytes (**Fig. 4A** and **Fig. S4A**). As major paracrine contributors, DAMPs reflect the senescence process of the cell-interaction community by their re-localization and secretion. Compared with the apparent up-regulated DAMPs genes mainly expressed in hepatocytes (hepatocyte-sequencing data), LSECs emerged the much more obvious senescence circumstance instead of DAMP release (LSEC-sequencing data). Notably, hepatocytes expressed partial pro-inflammatory SASP genes less than LSECs, whereas hepatocytes much earlier and easier released a mass of DAMPs than LSECs under HIRI insult (**Fig. 4B** and **Fig. S4B**).

Thus, we supposed that hepatocytes impact the SASP state of LSEC by releasing DAMPs much quicker, and further regulate the pro-senescence crosstalk among multiple cells under HIRI. Therefore, we then used a co-culture model insulted by HR *in vitro* to mimic intercellular communication between injured hepatocytes and SASP-LSECs (**Fig. 4C**). Interestingly, the expression of makers of senescence (*p21*, *p53*) and proinflammatory SASP (*cxcl1*) were significantly increased but cell cycle markers (*cdk2*, *ccnd1*, *cdc25*) were decreased in LSECs after treated with the conditional medium (CM) from injured hepatocytes insulted by HR, which also remarkedly reversed by ACT (**Fig. 4D**). Here, the apoptosis of LSECs was noticed in HR group but not after ACT intervention as evidenced by the flow cytometry (**Fig. 4E**). Later, we wanted to determine whether HMGB1 as typical DAMP participated in this cell-to-cell communication. Of noted, we roundly profiled the genes expression of DAMPs in hepatocytes and LSECs and found HMGB1 highly stuck out in these proinflammatory SASPs (**Fig. 4F**). Consistently, the visibly increased expression of HMGB1 was observed in serum and livers of HIRI mice, which were rapidly inhibited by ACT (**Fig. 4G**). To be more precisely, HMGB1 was prone to generated earlier and much more from hepatocytes than LSECs at the earlier stage of HR process as tested by ELISA and qPCR assay (**Fig. 4H**), more importantly, which could be inhibited by ACT treatment. Notably, the increased nuclear translocation and secretion of HMGB1 in hepatocytes during HR was also observed (**Fig. 4I** and **Fig. S4C**). At the same time, we also found that CM from HR-stimulated hepatocytes increased the levels and secretion of HMGB1 in LSECs and the uptake deficiency of FITC-FSA by fluorescein intensity analyzing, which were significantly decreased by different dosages of ACT (**Fig. 4J** and **Fig. S4C**). In addition, the level of HMGB1 and pathological markers of LSECs (NO and vascular endothelial growth factor A [VEGFA]) were increased after the HR medium stimulated and decreased by the ACT in LSECs (**Fig. 4K**). These data strongly pointed out that senescent LSECs under HR injury might not only suffer from direct environmental stimulus but also bear the secondary damage induced by a mass of HMGB1 released by a paracrine or autocrine manner from neighboring hepatocytes and LSECs themselves.

### HMGB1 counteracted ACT efficacy to aggravate the senescent of LSECs

**Figure 5:**
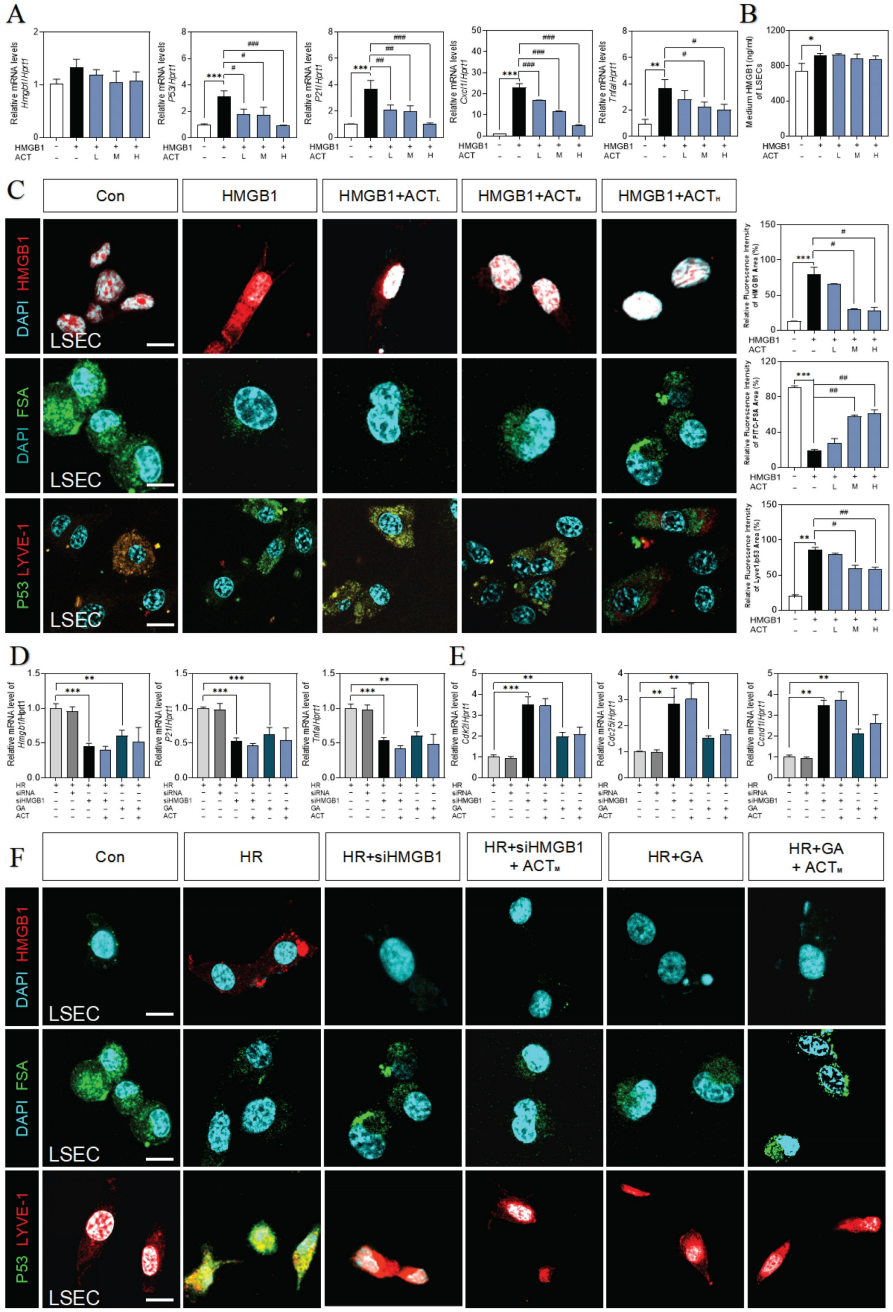
** ACT inhibited the HMGB1-induced senescence progression of LSECs.** (**A**) Relative expression of mRNA levels of *hmgb1*, *p53*, *p21*, *Cxcl1* and *tnfa* were measured by qPCR and further normalized with *hprt1* in LSECs with the treatment of recombinant HMGB1 protein and ACT. (**B**) ELISA results of HMGB1 in medium of LSECs. (**C**) Representative images of immunofluorescence staining for HMGB1, FITC-FSA, P53 and LYVE-1 of LSECs, scale bar = 100 μm. Relative mRNA levels of (**D**) *hmgb1*, *p21*, *tnfa*, (**E**) *cdk2*, *cdc25* and *ccnd1* were measured by qPCR and further normalized with *hprt1* in LSECs with siHMGB1 transfection or glycyrrhizin (GA) and ACT treatment. (**F**) Representative images of immunofluorescence staining for HMGB1, FITC-FSA, P53 and LYVE-1 of LSECs, scale bar = 100 μm. Statistical significance: *P < 0.05, **P < 0.01, ***P < 0.001, compared with the control group; ^#^*P* < 0.05, ^##^*P* < 0.01, ^###^*P* < 0.001 compared with the relative model groups.

Next, we applied recombinant HMGB1 protein to further confirm the correlation of secreted HMGB1 with SASP-LSEC transition. After being exposed to superfluous HMGB1 proteins, LSECs exhibited a trend of increased SASP-associated genes (increased *hmgb1*, *p21*, *p16*, *il6*, *p53*,* tnfa* and *cxcl1*, obvious cell cycle arrest with decreased *cdk2*,* cdk4,* and *ccnd1*), which were significantly revised by different dosages of ACT (**Fig. 5A** and **Fig. S5A**,** S5B**). Meanwhile, we found that the secretion of HMGB1 was also continuously increased to some extent in LSECs directly stimulated by HMGB1 (**Fig. 5B**). Subsequently, immunofluorescence assay shown in **Fig. 5C** confirmed that purified HMGB1 protein markedly induced the nuclear translocation of intracellular HMGB1 (the release of HMGB1 have been also considered as an indicator of cell senescence), promoted the loss of endocytosis ability (lower FITC-FSA uptake) and damaged typical fenestration phenotype (increased green dots but disrupted discontinuous-red dots) in HMGB1-stimulated group, while ACT effectively blocked these processes. Furthermore, we inhibited HMGB1 using siRNA or glycyrrhizin (a chemical inhibitor of HMGB1) in LSECs to confirm the driving role of exogenous HMGB1 on the LSEC senescence. As expected, siHMGB1 or glycyrrhizin strongly inhibited intercellular expression of HMGB1, accompanied with the decreased levels of SASP genes (*p21*, *tnfa*, *p53, il6*, *cxcl1*) and increased levels of cell arrest checkpoints (*cdk2*, *cdk4*, *ccnd1*) (**Fig. 5D**,** 5E** and **Fig. S5C**), as well as the remission of damaged function and structure of LSECs (improved FSA-uptake ability and senescence and fenestrated phenotype), which were not enhanced by ACT as well (**Fig. 5F**). Therefore, we demonstrated that exogenous HMGB1 induced the internal senescence procedure of LSECs and thus aggravated HIRI, which could be the key candidate target of ACT.

### ACT retarded LSEC aging by inhibiting the HMGB1-TLR4/3 axis

**Figure 6:**
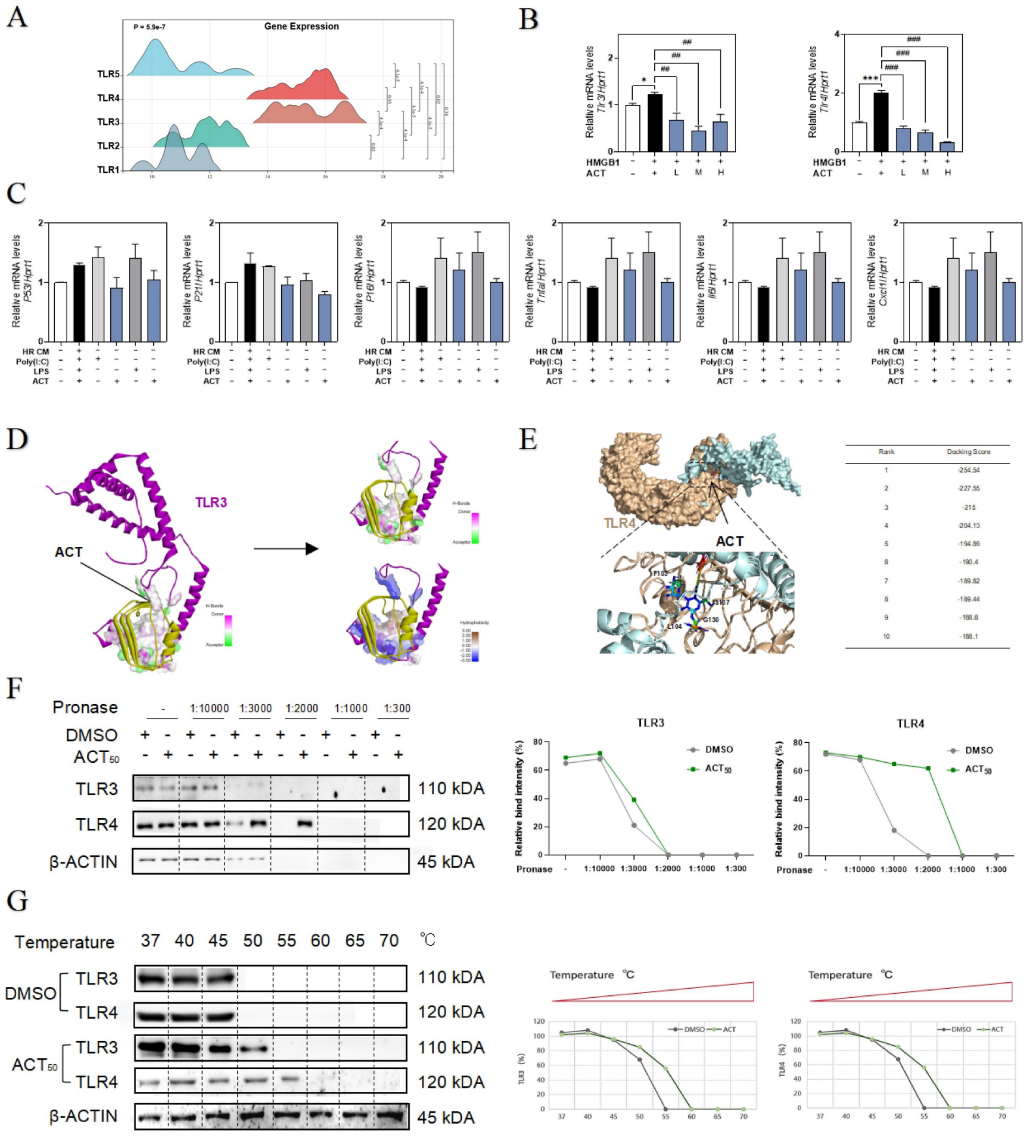
** ACT targeted HMGB1-TLR4/3 axis in LSECs.** (**A**) The ridge plot of TLRs in mRNA-sequencing analysis of primary LSECs. (**B**) Relative expression of mRNA levels of *tlr3* and *tlr4* were measured by qPCR and further normalized with *hprt1* in LSECs treated with recombinant HMGB1 protein and ACT. (**C**) Relative expression of mRNA levels of *p53*, *p21*,* p16*, *Tnfa*, *Il6* and* cxcl1* were measured by qPCR and further normalized with *hprt1* in LSECs treated with HR CM, Poly (I:C), LPS and ACT. Molecular docking site of ACT and the binding proteins (HMGB1 and TLR3 (**D**); HMGB1 and TLR4 (**E**)), and the ranking score of ACT binding sites. (**F**) The DARTS results of TLR3 and TLR4 in LSECs under different concentration of pronase and (**G**) the CETSA results of TLR3 and TLR4 in LSECs under different temperature was measured by western blot and normalized with β-actin. Statistical significance: *P < 0.05, ***P < 0.001, compared with the control group; ^##^*P* < 0.01, ^###^*P* < 0.001 compared with the HMGB1 group.

Following the depth bioinformatics analysis of RNA-sequencing of single cell type of primary LSECs, we noticed the high correlation of SASP genes with multiple TLRs that were also responsible for responding to various DAMPs including HMGB1 (**Fig. 6A**). Among these TLRs, *tlr3* and *tlr4* were significantly upregulated by recombinant HMGB1 protein and downregulated by ACT treatment (**Fig. 6B** and **Fig. S6A**).

To clarify the relevance between LSECs dysfunctional changes and TLR3/TLR4, the TLR3 agonist Poly (I:C) and TLR4 stimulator LPS were added in the CM of HR hepatocytes to treat LSECs, and as expected, the expression of* p53*, *p21*,* p16*, *tnfa*, *Il6* and* cxcl1* were all significantly increased by Poly (I:C) or LPS in LSECs, which were also inhibited by different dosages of ACT to some extent (**Fig. 6C**). For further verifying the subsequent intracellular TLR3/4 signal cascades that happened in LSECs following the HMGB1 release, the molecular docking analysis (**Fig. 6D** and** 6E**, **Fig. S6A** and** 6B**) showed the stabilized complexes and interaction between the crystallographic structure of HMGB1 protein (PDB code: 6CIK) and TLR3 (PDB code: 2Z1G) and TLR4 protein (PDB code: 4G8A). Meanwhile, as illustrated in **Fig. 6F**, the DARTS assay, which directly identified the effect targets for the ligand without additional modification, was employed for identifying the promising receptors binding with ACT. We incubated LSECs lysates with 50 μM ACT and found that the stability of TLR3 and TLR4 was enhanced *via* binding with ACT. In addition, the interaction between ACT and TLR3/4 was further demonstrated by CETSA method. As expected, ACT incubation stabilized TLR3/4 in the heat denatured LSECs lysates (**Fig. 6G**). These above results conjointly illustrated that TLR4 and TLR3 in LSECs were provoked by the extracellular HMGB1 to lead or aggravate the SASP status of LSECs, whereas this aging trend exacerbated by TLR4 and TLR3 might be a promising candidate target for ACT.

### ACT blocked LSECs senescence *via* disrupting the IRF1 transcription

**Figure 7:**
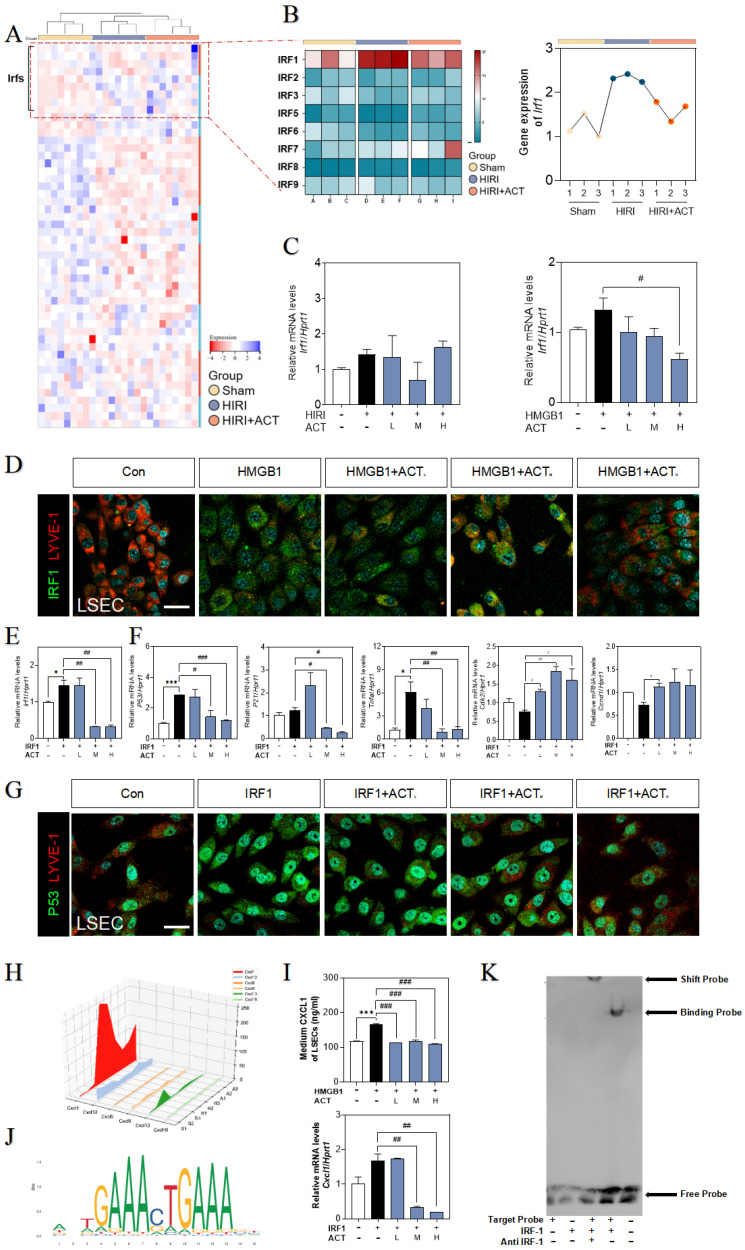
** The recombinant IRF1 protein promoted the senescence of LSECs and could be blocked by ACT.** (**A**) The heatmap of transcription factors in the whole mice liver. (**B**) The heatmap and tendency chart of IRFs in the whole mouse liver from Sham, HIRI, and ACT group. Relative expression of mRNA levels of *irf1* (**C**,** left panel**) in mouse liver under HIRI and ACT treatment and (**C**,** right panel**) in LSECs under recombinant HMGB1 protein and ACT treatment were measured by qPCR and further normalized with *hprt1*. (**D**) Representative images of immunofluorescence staining for IRF1 and LYVE-1 of LSECs with recombinant HMGB1 protein and ACT treatment, scale bar = 100 μm. Relative expression of mRNA level of (**E**) *irf1*, (**F**) *p53*, *p21*, *tnfa*,* cdk2* and* ccnd1* were measured by qPCR and further normalized with *hprt1* in LSECs with IRF1 and ACT stimulation. (**G**) Representative images of immunofluorescence staining for IRF1 and LYVE-1 of LSECs with recombinant IRF1 protein and ACT stimulation, scale bar = 100 μm. (**H**) The three-dimensional graph of gene expression of CXCLs in primary LSECs. (**I**) ELISA results of CXCL1 in LSECs under HMGB1 stimulation and relative expression of mRNA levels of *cxcl1* were measured by qPCR and further normalized with *hprt1* in LSECs stimulated by IRF1 and ACT. (**J**) The predicted transcription sequence of IRF1. (**K**) The EMSA results of CXCL1-targeted probe of IRF1. Statistical significance: *P < 0.05, *P < 0.01, ***P < 0.001, compared with the control group; ^#^*P* < 0.05, ^##^*P* < 0.01, ^###^*P* < 0.001 compared with the relative model groups.

Given the effects of HMGB1-TLR3/4 on senescence LSECs, we hypothesized that HMGB1-TLR3/4 modulates the transcription of senescence-related genes under HIRI stimulation. Based on the RNA-seq data of primary LSECs, hierarchical clustering and GSEA of differentially expressed transcription factor genes after HIRI revealed the notable upregulation of IRF1 (**Fig. 7A** and **Fig. S7A**). Consistently, the visualized fluctuation of IRFs gene maps of the HIRI liver were consistent with the results of RNA-sequencing expression profiles, especially for hepatic *Irf1* levels in livers (**Fig. 7B** and** Fig. 7C**,** left panel**). Also, LSECs incubated with recombinant HMGB1 protein showed the high expression of IRF1 (**Fig. 7C**), accompanied by the fenestration damage (marked by the dimer red laser of LYVE-1) (**Fig. 7D** and **Fig. S7B**), which could be effectively alleviated by ACT. In addition, the changes of SASP-related genes *irf1*, *p53*, *p21*, *tnfa*, *il6*, *Icam1*, *cdk2*,* ccnd1* and *cdc25* in LSECs treated by recombinant IRF protein were also blocked by ACT (**Fig. 7E**,** 7F** and **Fig. S7C**). Meanwhile, the loss of fenestration phenotype in IRF1-treated LSECs was also observed through immunofluorescence assay and reversed by ACT (**Fig. 7G** and **Fig. S7D**). We further analysis the expression of downstream CXCLs for evaluating role of IRF1 in these paracrine proinflammatory SASP cytokines of primary LSECs. As shown in** Fig. 7H**, compared with different chemokines based on RNA-sequencing data from primary LSECs, CXCL1, a member of chemokines affected in HIRI process, was highly noted. ELISA results showed the levels of CXCL1 in CM secreted by LSECs either treated with recombinant HMGB1 or IRF1 proteins but not in ACT groups were significantly increased (**Fig. 7I**). Furthermore, CXCL1 functioned as a potential affected chemokine neared the transcription start site of IRF1-dependent (**Fig. 7J** and **Fig. S7E**). As expected, EMSA results (**Fig. 7K**) showed the specific binding property mutation of CXCL1 and IRF1. This evidence confirmed that IRF1, as an interesting senescent candidate, accelerated the aging process and proinflammatory SASP of LSECs under the modulating of HMGB1-TLR3/4 but could be effectively inhibited by ACT.

### ACT inhibited the CXCL1-restraint crosstalk between SASP-LSECs and neutrophils

**Figure 8:**
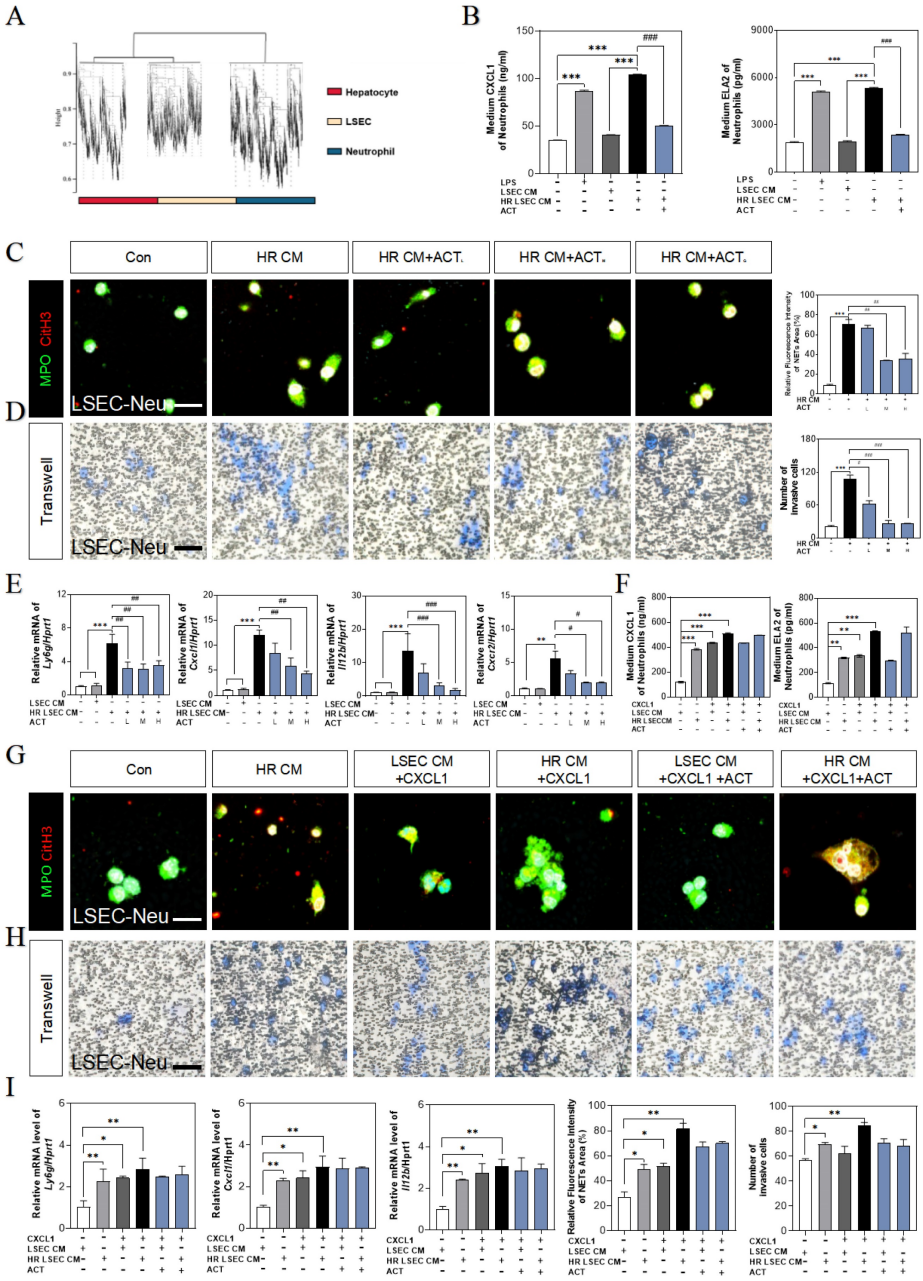
** ACT inhibited LSECs senescence and NETs formation by a CXCL1-restraint manner.** (**A**) The cell hallmarks analysis of mouse liver. (**B**) ELISA results of CXCL1 and ELA2 in the CM of LSECs. (**C**) Representative images of immunofluorescence staining for MPO and citH3 of primary neutrophils treated by different LSECs CM, scale bar = 100 μm. (**D**) Representative transwell images of primary neutrophils treated by different LSECs CM, scale bar = 60 μm. (**E**) Relative mRNA levels of *ly6g*,* Cxcl1*, *Il12b* and *Cxcr2* in neutrophils under LSECs CM and ACT treatment were measured by qPCR and further normalized with *hprt1*. (**F**) ELISA results of CXCL1 and ELA2 in the CM of primary neutrophils stimulated by recombinant CXCL1 protein, CM from HR LSECs and ACT. (**G**) Representative images of immunofluorescence staining for MPO and citH3 of primary neutrophils cultured with LSEC CM, recombinant CXCL1 protein and ACT treatment, scale bar = 100 μm; (**H**) Representative transwell images of primary neutrophils, scale bar = 60 μm. (**I**) Relative mRNA levels of *ly6g*,* cxcl1* and *il12b* in neutrophils stimulated by recombinant CXCL1 protein, CM from HR LSECs and ACT were measured by qPCR and further normalized with *hprt1*. Statistical significance: *P < 0.05, *P < 0.01, ***P < 0.001, compared with the control group; ^##^*P* < 0.01, ^###^*P* < 0.001 compared with the model group.

We also noticed that a plenty of immune cells were enriched in hepatic sinusoid, especially surrounding aged LSECs (**Fig. 1**), and consistently, neutrophils were significantly increased in RNA-sequencing data of the whole liver (**Fig. 8A**). In order to clarify the consequences of the abundant release of CXCL1 from aged LSECs or experimental HIRI, we isolated and explored whether the function and phenotype of primary neutrophils had any changes after treated by CM of HR LSECs or LPS (an activator of neutrophils) (**Fig. S8A** and** S8B**) and found the increased levels of CXCL1 and ELA2 (also known as neutrophil elastase, NE) in activated neutrophils but not in ACT-treated neutrophils (**Fig. 8B**). Correspondingly, there was also a clear formation of neutrophil extracellular traps (NETs) marked by MPO and CitH3 (manifested by the extracellular filiform structure), after treated with CM from SASP-LSEC but reversed by CM containing ACT (**Fig. 8C**). We also constructed a transwell system (primary LSECs were loaded in the upper chamber and primary neutrophils were seeded in the plates) and clearly showed the evident migration of neutrophils after incubated with SASP-LSEC but not ACT-treated LSEC (**Fig. 8D**).

Meanwhile, the gene expressions of neutrophils maker (lymphocyte antigen 6 complex locus G, *ly6g*) and NETs makers (*cxcl1*, *Il12b*, *cxcr2*) were highly increased after incubated with CM from HR-stimulated LSECs, while ACT-pretreated neutrophils displayed lower recruitment and NETs formation (**Fig. 8E**). For better evaluating the role of CXCL1 in neutrophils recruitment and the beneficial effects of ACT on NETs formation during HIRI, recombinant CXCL1 protein was applied in this co-cultured system, as expected, the levels of CXCL1 and ELA2 were up-regulated in CXCL1-stimulated CM from SASP-LSECs and could not be protected by ACT anymore (**Fig. 8F**).

In addition, the NETs formation marked by the co-staining of MPO and CitH3 (**Fig. 8G**), the invasive cell numbers of primary neutrophils (**Fig. 8H**) and the gene expressions of *ly6g*, *cxcl1* and *il12b* (**Fig. 8I**) were more significantly upregulated in recombinant CXCL1 protein-treated primary neutrophils than those only incubation with CM from HR-stimulated LSECs, while ACT showed lightly inhibitory effects on relative above process. These data suggested that the formation of NETs was dependent upon IRF1-mediated CXCL1 under HIRI, which could be alleviated by ACT pretreated.

### IRF1-overexpressed LSEC-targeting liposomes neutralizes ACT effects better than HMGB1 stimulation

**Figure 9:**
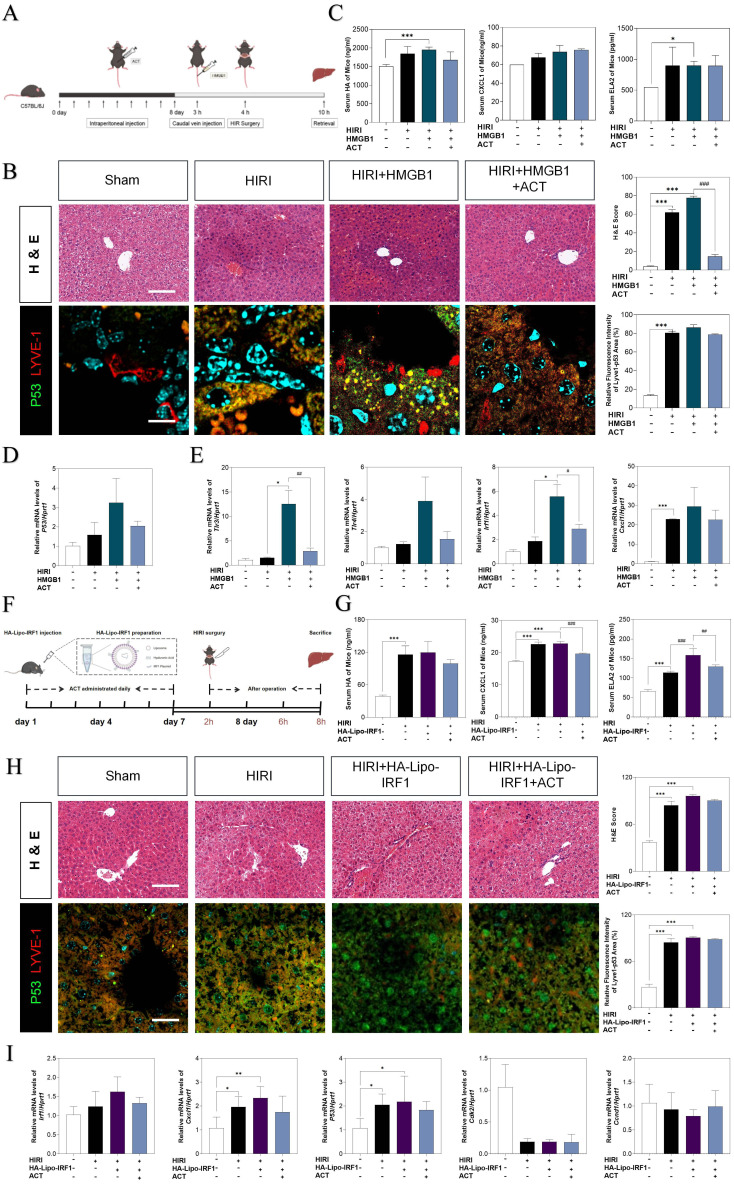
** The overexpression of HMGB1 or IRF1 reversed therapeutic effects of ACT.** (**A** and **F**) Flow chart of animal experiment. (**B**) Representative images of H & E staining (scale bar = 20 μm) and immunofluorescence staining for P53 and LYVE-1 (scale bar = 100 μm) in mouse livers treated with recombinant HMGB1 protein and ACT. (**C**) ELISA results of HA, CXCL1 and ELA2 in mouse serum treated with recombinant HMGB1 protein and ACT. (**D**) Relative mRNA levels of *p53*,* (***E***) tlr4*,* tlr3*, *irf1* and* cxcl1* were measured by qPCR and further normalized with *hprt1* in mouse livers. (**G**) ELISA results of HA, CXCL1 and ELA2 in the mouse serum treated with HA-Lipo-IRF1 and ACT. (**H**) Representative images of H & E staining (scale bar = 20 μm) and immunofluorescence staining for P53 and LYVE-1 (scale bar = 100 μm) in mouse livers treated with HA-Lipo-IRF1 and ACT. (**I**) Relative mRNA levels of *irf1*,* cxcl1*,* p53*,* cdk2* and *ccnd1* were measured by qPCR and further normalized with *hprt1* in the mouse livers treated with HA-Lipo-IRF1 and ACT. Statistical significance: *P < 0.05, *P < 0.01, ***P < 0.001, compared with the sham group; ^#^*P* < 0.05, ^##^*P* < 0.01, ^###^*P* < 0.001 compared with relative model groups (n = 6).

To further corroborate the role of HMGB1-mediated LSECs senescence in HIRI, the recombinant HMGB1 protein was intravenously injected from tail vein before HIRI surgery and ACT pre-administration in mice (**Fig. 9A**). Herein, histological examination (**Fig. 9B**), serum HA, CXCL1 and ELA2 levels, hepatic function indexes (AST, ALT, LDH, NO in the serum and MDA, SOD, NO in the liver) (**Fig. 9C**, **Fig. S9A**, **S9B** and **S9C**) analyses indicated that the degree of sinusoidal congestion and ischemic section, LSECs senescence and fenestration lesion were aggravated under the HMGB1 stimulation and alleviated upon ACT pretreatment to some extent. Besides, the expression of *hmgb1*, *klf2*, *p53*, TLRs (*tl3*, *tlr4*) and neutrophils recruiters (*irf1*,* cxcl1*) were upregulated after HMGB1 activation, which were slightly downregulated by ACT as well (**Fig. 9D**,** 9E** and **Fig. S9D**). For further evidence the role of IRF1 played in HIRI, the HA-Lipo-IRF1 was prepared for directly targeting to IRF1-overexpressed LSECs and injected into the HIRI mice before ACT treatment (**Fig. 9F** and **Fig. S9E**). Similarly, the levels of HA, CXCL1, ELA2 and hepatic function indicators in serum and liver demonstrated the function and secreted-phenotype of LSECs were injured by the superfluous IRF1 induced by LSECs-specified HA-Lipo-IRF1 plasmid (**Fig. 9G** and **Fig. S9F**). In addition, compared with the HIRI group, the pale and ischemic region showed in H & E staining and the fluorescence co-staining of P53 and LYVE-1, IRF1 and LYVE-1 were both aggravated in the HA-Lipo-IRF1 group and only slightly changed after ACT intervention (**Fig. 9H** and **Fig. S9G**). Meanwhile, the expression of *Irf1*, *cxcl1* and *p53* were increased, and the cell-cycle checkpoints (*cdk2* and* ccnd1*) were downregulated by the infusion of HA-Lipo-IRF1, while ACT failed to intercept the progress of LSECs senescence at this time (**Fig. 9I**). These data demonstrated that the overexpression of HMGB1 or LSECs-specific overexpressed IRF1 could directly reverse the therapeutic effects of ACT on HIRI mice to different degrees.

## Discussion

Accumulating evidence showed that the hepatic sinusoid microenvironment plays an essential role in the liver ischemia-reperfusion process 32, 33, including maintaining the integrity and functionality of endothelial system in terminal vascular unit, the nutrient exchange and immunologic balance of the unique liver microcirculatory system. We noticed a basis of senility-oriented cell cycle abnormalities of LSECs and DAMP-secreted cellular stress response during the HIRI (**Fig. 1** and **2**). However, much less is known concerning what irritates and links up the DAMPs-secreted hepatocytes and senescent LSECs in the HIRI progression (**Fig. 3**), which offers a valuable priority for our current studies. Hereon, by applying the direct evidence of molecular biology and sequencing bioinformatics, we confirmed how HMGB1 from injured hepatocytes drived LSECs senescence phenotypes (**Fig. 4** and **5**), led the activation and binding with membrane receptor TLR4 and intracellular transferable receptor TLR3 (**Fig. 6**), which induced the nuclear translocation of the transcription factor IRF1 and consequent excess paracrine pro-inflammatory SASP (**Fig. 7**), subsequently recruited neutrophils to form NETs and aggravated hepatic sinusoidal injury (**Fig. 8**).

Importantly, we firstly determined that ACT elicited the senescence of LSECs induced by hepatocytes-secreted HMGB1 and thus improved the hepatic sinusoid microenvironment under the HIRI process through disturbing the HMGB1-TLR4/3-IRF1 signaling, nevertheless, the exogenous injected HMGB1 and/or LSECs-specific over-expressed IRF1 could directly blockade the protective effects of ACT (**Fig. 9**).

The cellular-senescence occurs on diversified hepato-cell populations during HIRI progression especially for aging LSECs, as demonstrated by the tendentious RNA-sequencing analyses and altered cellular phenotype including the increased SA-β-Gal^+^ staining, reduced EdU incorporation and upregulated p53 and p21 and consistent G1/S cell cycle arrest, that can contribute to further inflammation and tissue injury (**Fig. 3**). Moreover, LSECs, as a very specialized fenestrated endothelial character and immune gatekeeper of the hepatic sinusoid, were characteristically loose the phenotype of fenestration and injured endocytosis ability after HIRI, and failed to maintain the liver hemodynamics, sinusoids remodeling and wall shear stress. Notably, we found that ACT not only directly reverse the phenotype of senescent LSECs, but also modified the liver sinusoid microenvironment, which remind us ACT might also take part in the crosstalk between LSECs and others damaged hepatic cells under HIRI. Initially, HMGB1 is usually firstly secreted by hepatocytes, due to the dominant number of hepatocytes and the vulnerability to be impacted largely in the whole liver 34. Recently, it has been reported that the nuclear translocation of HMGB1 could drive intracellular senescence 35. However, whether HMGB1 could drive paracellular senescence as a paracrine proinflammatory SASP is still unclear. Of noted, RNA-sequencing data of single cell type of hepatocytes provided interesting evidence that HMGB1-dominated DAMPs from hepatocytes were obviously increased and released into the extracellular environment, which correlated with upregulated senescent genes in LSECs, suggesting that HMGB1 might contribute to the process of senescence taken place in the LSECs (**Fig. 4B**,** 4D** and **4H**). Therefore, we hypothesized that HMGB1 mainly secreted by hepatocytes induced and exacerbated the consequent senescence of LSECs. More importantly, we also provided evidence that post-transcriptional gene silencing of HMGB1 ameliorated the transition of LSECs into aging phenotype and deactivated the proinflammatory SASP, which could be enhanced by ACT administration (**Fig. 5C**-**5E**). Therefore, the interaction between injured hepatocytes and LSECs through HMGB1 release and clearance is a critical mechanism in the pathophysiology of HIRI. Understanding the complex interactions between these two cell types may offer new insights into the discovery of therapeutic strategies targeting HMGB1 release and clearance to prevent or mitigate the damaging effects of HIRI.

TLRs are involved in a wide range of intracellular signal transduction and nuclear translocation, as well as a kind of well-accepted HMGB1 receptors and transcription factor closely related to cellular proliferation, apoptosis, and senescence 36. Generally, HMGB1 could activate and binding with TLR4 and TLR3, especially the preponderant cell-membrane receptor TLR4 37, 38. Here, we found that hyper-expressive TLR3 and TLR4 facilitated the LSECs' aging trend and functional loss using Poly (I:C) and LPS as agonists of TLR3 and TLR4. Furthermore, the molecular docking and immunoblot assay (DARTS and CETSA) indicated that ACT might slow down the senescence process of LSECs by interfering with the binding site of HMGB1-TLR3/4 to inhibit the consequent nuclear translocation (**Fig. 6F** and** 6G**). Besides, site mutation and docking data proved that lys730 of TLR4 was important for the interaction with ACT to influence the senescence of LSECs. We noticed that HR treatment potentiated TLR3 and TLR4 to enhance the proinflammatory-SASP genes expression (**Fig. 6C**). In fact, TLR4 and TLR3 well-established a partnership of coordination in leading the production of a wide range of immune stimulatory cytokines and chemokines, including the generation of SASP genes 39. A number of studies reported that, as the first identified TLRs, TLR4 at the plasma membrane has been emphasized for recognizing the extracellular structural and nonstructural proteins to induce the transcription of cytokines and chemokines *via* the activation the TIR domain containing adaptor molecule 1 (TRIF)-related adaptor molecule, while it was noting that TLR3 was reported to be the only TLR to activate the TRIF until now 40, 41. However, in consideration of the docking intensity and the results of DARTs and CETSA, TLR4 and TLR3 still showed a closed combining capacity with ACT, therefore, ACT might exert the protective effect more inclined to rely on the collaboration of TLR4 and TLR3. Hence, we speculate that under the stimulation of exogenous HMGB1, the endosome TLR3 may tend to activate the TRIF pathway to assist the transmembrane TLR4 for more efficiently carry out the cellular signal transduction but further research is still needed.

After stimulating by extracellular HMGB1 from hepatocytes, the proinflammatory-SASP genes were markedly upregulated in SASP-LSECs. Recently studies showed that IRF1, IRF3 and IRF7 could be provoked in the cellular senescence 42, 43. Our sequencing data and *in vitro* study support the upregulation of IRF1 after either HIRI insult or TLR3/4 activation. In addition, the upregulation of IRF1 not only worsened the LSECs senescence and phenotypic dysregulation but also promoted the production and release of pro-inflammatory SASP from LSECs, which accelerated the inflammation happened in liver sinusoid and allowed IRF1 to be a signal for activating the followed immune cascade through controlling the proinflammatory SASP (**Fig. 7**). Moreover, senescence usually accelerated after sensing the abundant paracrine SASP release, which exerted the potential for various beneficial or even detrimental paracrine effects on liver self-regulation 44, 45. Notably, as a broad-spectrum transcriptional factor, IRF1 could also participate in the transcription of HMGB1 to release to the extracellular environment 46, suggesting that IRF1 may regulate the generation and secretion of HMGB1 to form an aggravated HMGB1-released original feedback in the progression of LSECs senescence. More importantly, our sequencing data supported that the most changed chemokine, CXCL1, was the downstream therapeutic target for improving HIRI, which was gradually demonstrated to combining with IRF1 by the promoter site prediction and EMSA analysis verification (**Fig. 7J** and** 7K**). Notably, IRF1 promoted the senescence of LSECs in a CXCL1-dependent transcription manner, which could be inhibited by ACT. In particular, CXCL1 has been recognized as a classical chemokine released from immune cells for promoting peripheral neutrophilic recruitment and NETs formation. In recent years, neutrophil migration and NETs formation have been gradually emphasized and highlighted in HIRI 15, 47. The neutrophils markers detection, transwell results, and MPO/citH3 co-localization showed that neutrophils have been activated and recruited to form the NETs during the HIRI progress, which could also be more easily observed after CXCL1 recombinant protein stimulation, suggesting that IRF1-dependent CXCL1 plays a unique role in promoting LSECs senescence and exacerbating the immune balance of hepatic sinusoid microenvironment.

Eventually, although ACT was effective in alleviating senescent phenotype of LSECs, the specific acting site of ACT in preventing SASP-gene transcription remains to be confirmed, especially the candidate sites, HMGB1 and IRF1. On the one hand, the recombinant HMGB1 protein exerts a partly inhibitive effect on ACT treatment instead of entirely inhibition, which suggested that HMGB1 might be the one of numerous upstream targets, but ACT might widely block the in-depth transcript sites to restrain SASP-LSECs. An increasing number of researches about cell-specific gene targeting based on liposome system were reported either by targeting ALB in hepatocytes 48 or and conjugating vitamin A in hepatic stellate cells 49. On the other hand, considering the importance of cell-targeted therapy, we also conjugated the LSECs-specific endocytic HA with the IRF over-expressed liposome for construct a LSECs-specific targeting system, and investigate whether the caudal injection of HA-Lipo-IRF1 could block the hepatoprotective effects of ACT. Interestingly, compared with the injected HMGB1 stimulation, we noticed that HA-Lipo-IRF1 more direly prevented the therapeutic effect of ACT on HIRI mice (**Fig. 9**). IRF1 broadly initiated the transcription of both pro-inflammatory SASP genes and HMGB1-donated DAMPs, which more likely and widely exacerbate the paracrine- and autocrine- SASP to impact the 'gatekeeper' role of LSEC in HIRI livers. Moreover, we have to say that the plasmid of specific IRF1 over-expression system is still an ongoing challenge. The conjunction of HA-derivative and Lipo-IRF1 plasmid was not easy to compose under a severe environment controller system, including the subtle mixability and filtration condition. Since the acknowledged LSECs-specific targeting system was not well-constructed up to now, the discovery of highly specific and effective therapy of IRF1 inhibitory combined with LSECs-specific endocytic vector still needs to be explored.

## Conclusion

In summary, we first elucidated the senescence characteristics of LSECs in the process of HIRI and provided ACT, a unique drug that interferes with TLR3/HMGB1 or IRF1 binding sites to alleviate HIRI injury and aging process. Targeting LSEC senescence and regulating the hepatic sinusoidal microenvironment like ACT may provide new approaches for preventing and treating HIRI.

## Supplementary Material

Supplementary methods, figures and table.

## Abbreviation

ACT: acteoside
CCND1: cyclin D1
CDC25: cell division control protein 25
CDK2: cyclin-dependent kinase 2
CitH3: citrullinated H3
CXCL1: chemokine (C-X-C motif) ligand 1
DAMPs: damage-associated molecular patterns
HA: hyaluronic acid
HIF1α: hypoxia-inducible factor 1 subunit alpha
HIRI: hepatic ischemia-reperfusion injury
HMGB1: high mobility group protein 1
HNF4α: hepatocyte nuclear factor 4
HR: hypoxia and reoxygenation
IRF1: interferon regulatory factor 1
LSEC: liver sinusoidal endothelial cell
LYVE-1: lymphatic vessel endothelial hyaluronic acid receptor
NAC: N-acetylcysteine
NO: nitric oxide
P16: cyclin dependent kinase inhibitor 2A
P21: cyclin dependent kinase inhibitor 1A
P53: tumor protein p53
ROS: reactive oxygen species
SASP: senescence-associated secretory phenotype
TLR: toll-like receptor
TNF-α: tumor necrosis factor-α
