# Supplementary material for: Acteoside ameliorates hepatic ischemia-reperfusion injury via reversing the senescent fate of liver sinusoidal endothelial cells and restoring compromised sinusoidal networks
Source: Int J Biol Sci. 2023 Sep 18;19(15):4967–88. doi: 10.7150/ijbs.87332 (PMC10539705; doi:10.7150/ijbs.87332)
Supplement: Supplementary file 1 — Supplementary methods, figures and table. [file ijbsv19p4967s1.pdf]

Supplementary Materials and Methods

1. Supplementary methods

1.1 Serum biochemical measurements

The blood of mice was collected from the inferior vena cava and was centrifuged for 10 min at 1200g to obtain serum. Alanine aminotransferase (ALT), aspartate aminotransferase (AST), lactic dehydrogenase (LDH), NO, and hyaluronic acid (HA) in the serum were measured by relative detection kits to evaluate the degree of HIRI in mice, according to the detailed manufacturer's specifications.

1.2 Histopathological examination

After being fixed in 4% paraformaldehyde and embedded in paraffin, liver specimens stained with hematoxylin and eosin (H & E) reagents were scanned and analyzed by super-resolution microscopy (Leica, Wetzlar, Germany). For transmission electron microscope (TEM) examination, liver tissues were immersed in specialized TEM formalin-free fixative solution, which was rapidly sliced and prepared for scanning to examine the hepatic sinusoid architectures. All images were recorded using Zeiss Libra 120 transmission electron microscopy (Carl Zeiss, Germany).

1.3 RNA isolation and quantitative RT-PCR.

The total RNA from different cells or HIRI mouse livers were isolated by TRIzol reagent and were reverse transcribed to cDNA. The expressions of the target genes were detected by SYBR Green quantitative real-time polymerase chain reaction. Target gene expressions were calculated by their ratios to the housekeeping gene Hprt1. Further inquiries of primers for qPCR can be directed to the corresponding

author.

1.4 Enzyme-linked immunosorbent assays (ELISA)

After setting up the standard curve, HMGB1 and C-X-C motif chemokine ligand 1 (CXCL1) from the cell culture medium and mouse serum was measured by different HMGB1 and CXCL1 ELISA kits, according to the described manufacturer's protocols. The optical density of each well was determined using a microplate reader.

1.5 Immunofluorescence staining

After fixation and embedded in paraffin, the liver tissue from mice was cut into 5 μm thick sections for IF staining. For immunofluorescence, the slices were deparaffinized in xylene and dehydrated in ethanol and blocked with 0.2% Triton X-100-2.5% BSA-1× PBS-10% goat serum after subjecting to antigen retrieval solution. The primary antibodies against HNF4a (dilution 1:400), ALB (dilution 1:500), CD11 (dilution 1:150), Lyve-1 (dilution 1:400), CK7 (dilution 1:300), KI67 (dilution 1:300), MPO (dilution 1:400), citrullinated histone H3 (CitH3) (dilution 1:400), P53 (dilution 1:1000), HMGB1 (dilution 1:400), IRF1 (dilution 1:300), CXCL1 (dilution 1:1000), were respectively incubated overnight at 4°C. After washing, the sections were incubated with goat anti-mouse IgG (H+L) highly cross-adsorbed 488 secondary antibody or anti-rabbit IgG (H+L) 594 secondary antibody (Thermo Fisher Scientific, Waltham, USA) and stained with DAPI. After staining of cell nuclear, the sections were sealed with resin and further observed using Aperio Versa (Leica, Wetzlar, Germany).

1.6 Flow cytometry analysis

Cell proliferation and apoptosis of LSECs were measured using AV/PI kit

(Beyotime, Shanghai, China) following the manufacturer's instruction. Briefly, the primary LSECs were harvested by trypsin for AV/PI staining, then washed with PBS and re-suspended by 200 μl 1× Binding Buffer with 5 μl FITC Annexin V and PI for 20 min in the dark at room temperature. Finally, AV/PI signals were visualized by a CytoFLEX flow cytometer (Beckman Coulter, Pasadena, CA).

1.7 Senescence associated β-galactosidase staining (SA-β-Gal)

SA-β-Gal staining kit (G1580) was obtained from Solarbio (Beijing, China) and the pre-heated SA-β-Gal solution was used for staining of aging hepatocytes and LSECs. Briefly, different hepatic cells in a 6-well plate were fixed with β-Gal formaldehyde solution for 15 min, washed with 1× PBS 3 times, and stained for 12h with X-Gal solution 1 ml. The slides were subsequently washed and mounted for microscope scanning and Image J was used for staining quantification.

1.8 5-Ethynyl-2'-deoxyuridine staining (EdU) incorporation and staining

The EdU staining kit (APEx BIO, USA) was used for evaluating the proliferation of hepatocytes and LSECs. Firstly, the 10× EdU buffer dissolving in dimethyl sulfoxide diluted with culture medium at 4 mM was prepared. Then, the cells in different groups were cultured in a 6-well plate and incubated with EdU buffer at a concentration of 2 mM for 2 h. After being washed twice and fixed in 4% formaldehyde for 15 min at room temperature, cells were incubated with 0.3% Triton X-100 diluting in 3% bovine albumin-PBS for 15 min and washed twice again. For the EdU click reaction, cells were incubated with the working buffer (including the 1× EdU reaction buffer, $CuSO_4$, Cy3 azide, and 1× EdU buffer addictive) for 30 min in the darkness. After being

washed for 2 times, cells were embedded with DAPI and observed under the confocal laser scanning microscopy (Olympus FV3000, Tokyo, Japan).

1.9 Transwell assay

For the transwell experiment of neutrophils recruited by LSECs, a total of 1 × 10^6 primary isolated neutrophils were seeded in the upper chamber of the transwell systems (0.4 μm) with 250μl 1% FBS DMEM medium, and 1 × 10^6 attached LSECs were added in the bottom chamber under HR process and treated with ACT (25, 50 and 100 μM) with 500ul 1% FBS DMEM medium for 6 h. After incubating for 12 h, the cells in the bottom were removed slightly with a cotton swab, while the cells on lower filter surfaces were fixed by the 4% formaldehyde and then stained with the 0.1% crystal violet solution for 30 min. The number of migrated cells was counted by an optical microscope (Carl Zeiss AG, Oberkochen, Germany).

Supplementary figures and legends

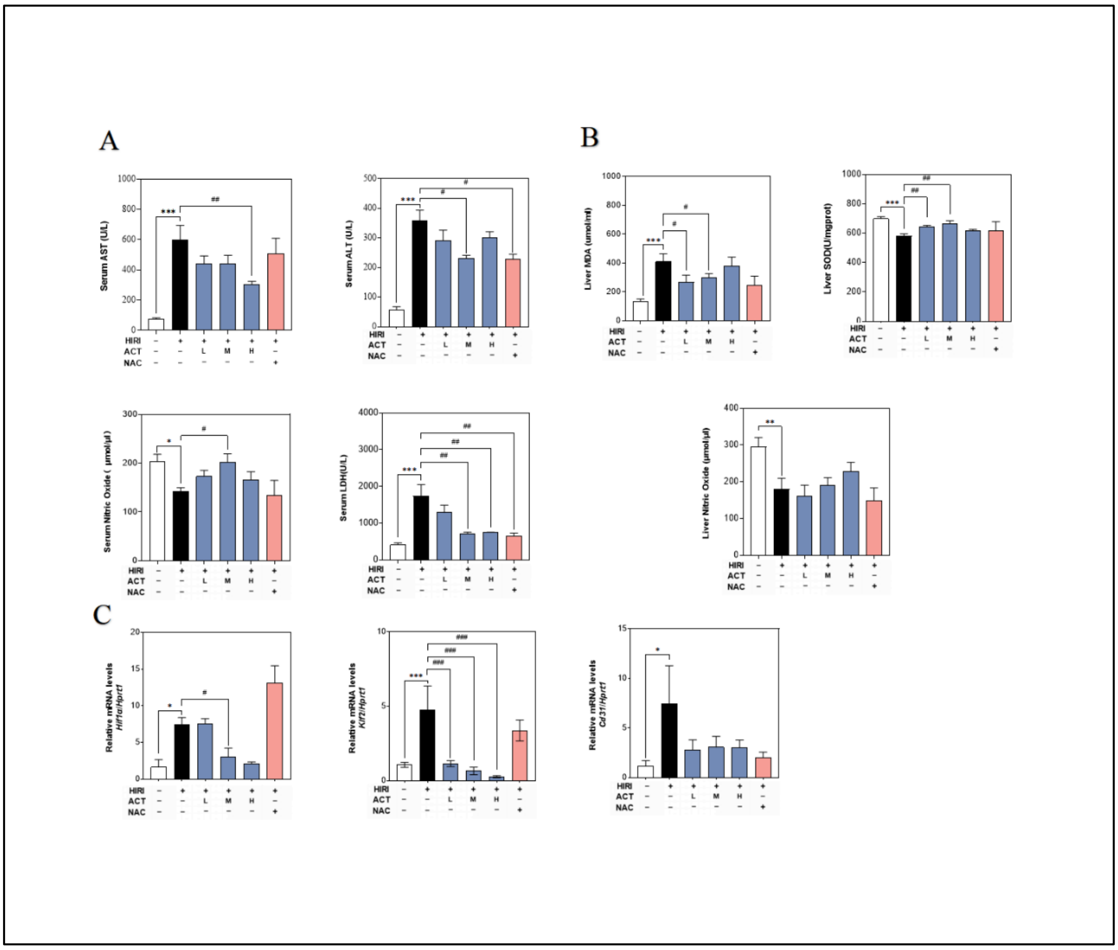

Fig. S1. ACT alleviates the hepatic function injured by HIRI process. (**A**) Serum levels of AST, ALT, NO and LDH. (**B**) The levels of MDA, SOD and NO in liver. (**C**) Relative expression of mRNA levels of *hif1a*, *klf2* and *cd31* were measured by qPCR and further normalized with *hprt1*. Statistical significance: **P* < 0.05, ***P* < 0.01, ****P*< 0.01; compared with the Sham group; #*P* < 0.05, ##*P* < 0.01, ###P< 0.01 compared with the HIRI group (n = 6).

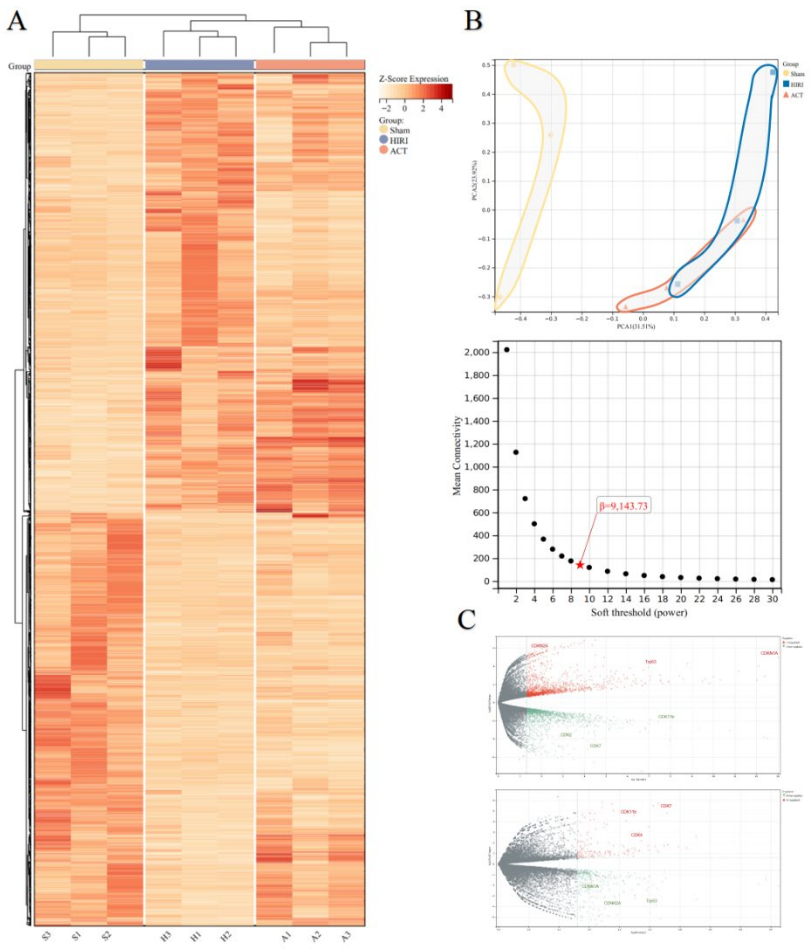

Fig. S2. SASP-related genes were up-regulated in HIRI group and down-regulated in ACT group. (**A**) The heatmap of whole genome in mouse liver from Sham, HIRI, and ACT group. (**B**) The PCA chart and soft threshold lines of enriched genes in different groups. (**C**) The volcano plot of enriched genes in different groups.

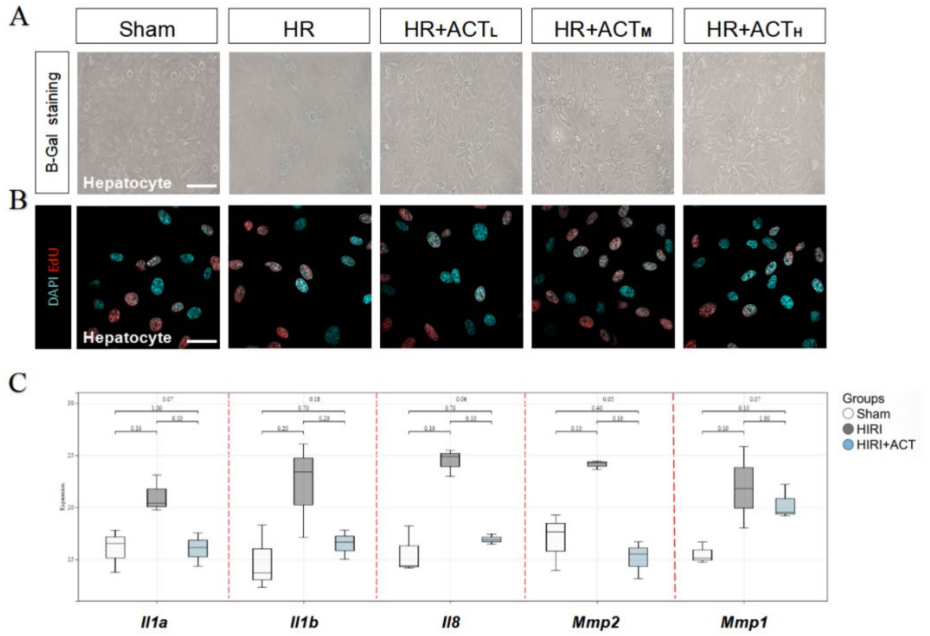

Fig. S3. Hepatocytes had no correlation with senescence and LSECs expressed proinflammatory SASP gene under HIRI. (**A**) The representative images of β-Galactosidase staining and immunofluorescence staining of EdU assay (**B**) of BRL (Scale bar = 100 μm). (**C**) The expression chart of representative SASP-related genes (*il1a*, *il1b*, *il8*, *mmp2*, *mmp1*) in mRNA-sequencing analysis results.

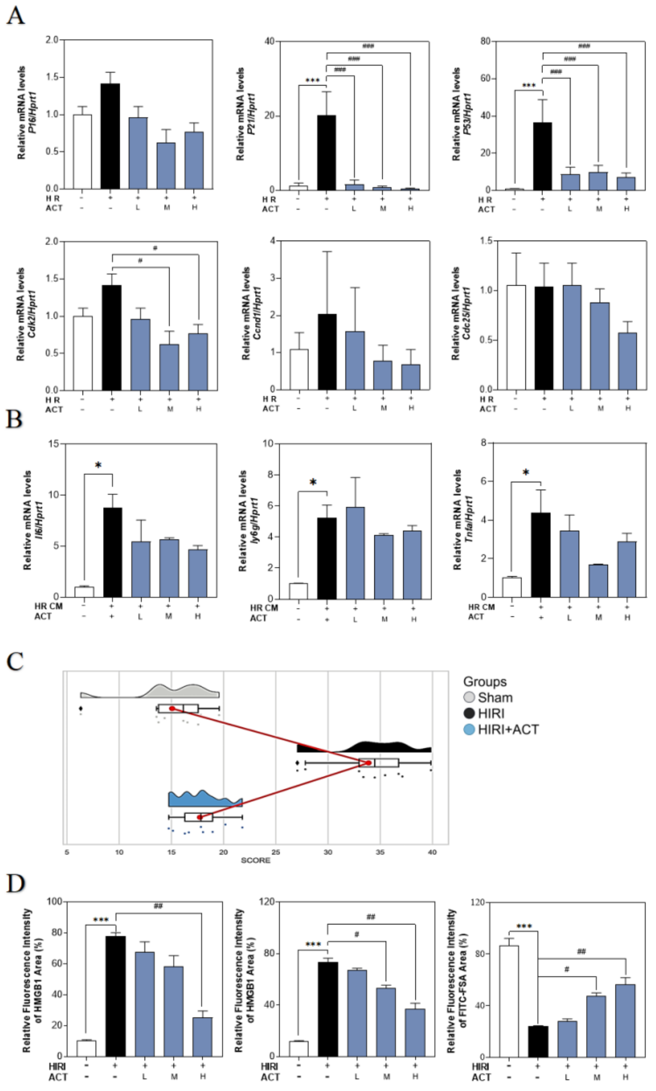

Fig. S4. LSECs exhibited evident senescent and decline phenotype. (**A**) Relative expression of mRNA levels of *p16*, *p21*, *p53*, *cdk2*, *ccnd1* and *cdc25* of BRL under HR insult were measured by qPCR and further normalized with *hprt1*. (**B**) Relative expression of mRNA levels of *il6*, *ly6g*, and *tnfa* of LSECs under HR CM insult was measured by qPCR and further normalized with *hprt1*. (**C**) The raincloud plot of HMGB1 gene of mouse liver in mRNA-sequencing analysis results. (**D**) The statistical charts of immunofluorescence staining of HMGB1 and FITC-FSA of hepatocytes and

LSECs. Statistical significance: $^{*}P < 0.05$; compared with the Con group; $^{\#}P < 0.05$, $^{\#\#}P < 0.01$, $^{\#\#\#}P< 0.01$ compared with the HR group; $^{***}P< 0.01$; compared with the Sham group; $^{\#}P < 0.05$, $^{\#\#}P < 0.01$ compared with the HIRI group (n = 6).

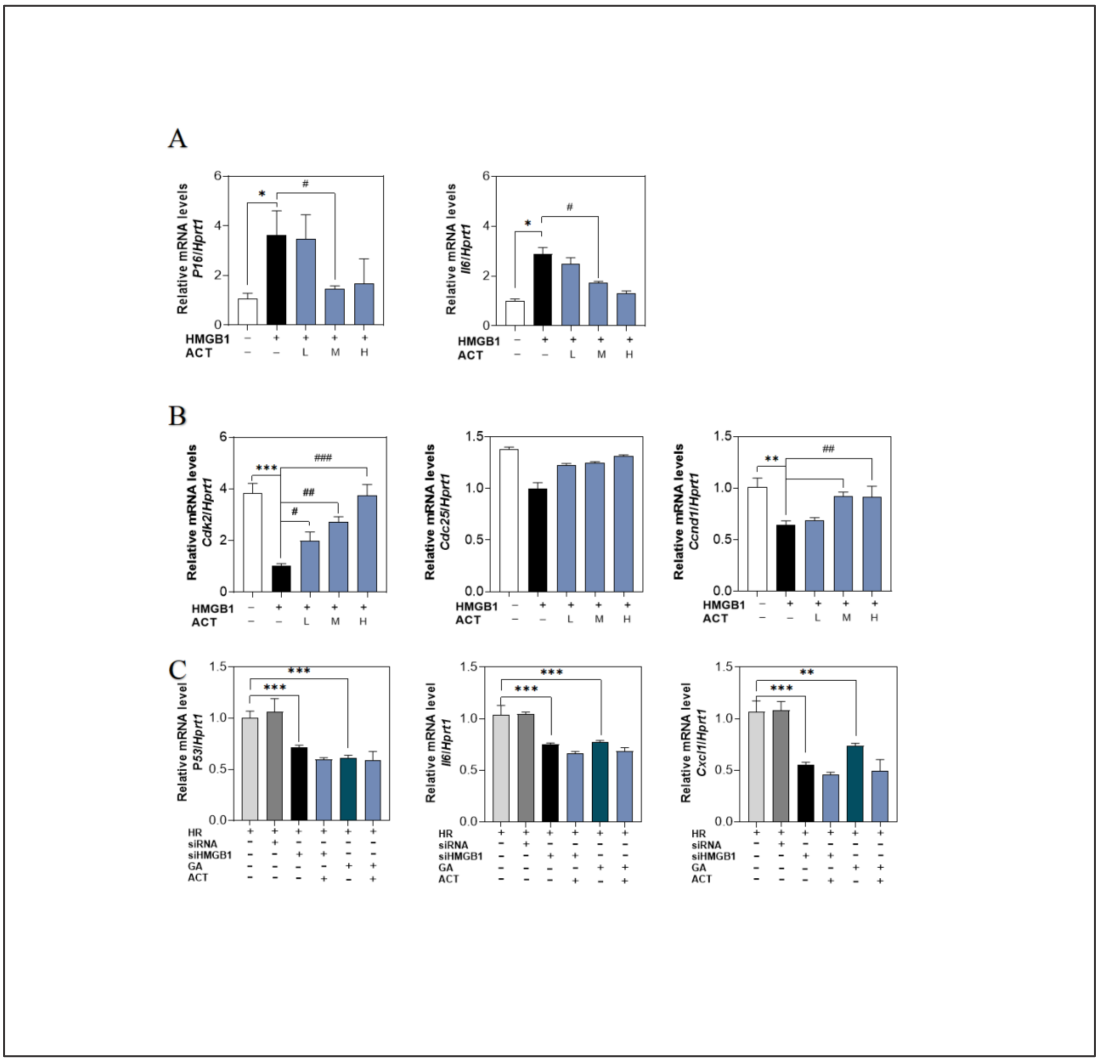

Fig. S5. The exogenic HMGB1 aggravated the senescence of LSECs. (**A**) Relative expression of mRNA levels of *p16*, *il6* and (**B**) *cdk2*, *ccnd1* and *cdc25* of LSECs under HMGB1 stimulation were measured by qPCR and further normalized with *hprt1*. (**C**) Relative expression of mRNA levels of *p53*, *il6*, and *cxcl1* of LSECs under siHMGB1 treatment were measured by qPCR and further normalized with *hprt1*. Statistical

significance: *$P < 0.05$, **$P < 0.01$, ***$P < 0.01$; compared with the Con group; #P < 0.05, ##$P < 0.01$, ###$P < 0.01$ compared with the HMGB1 group.

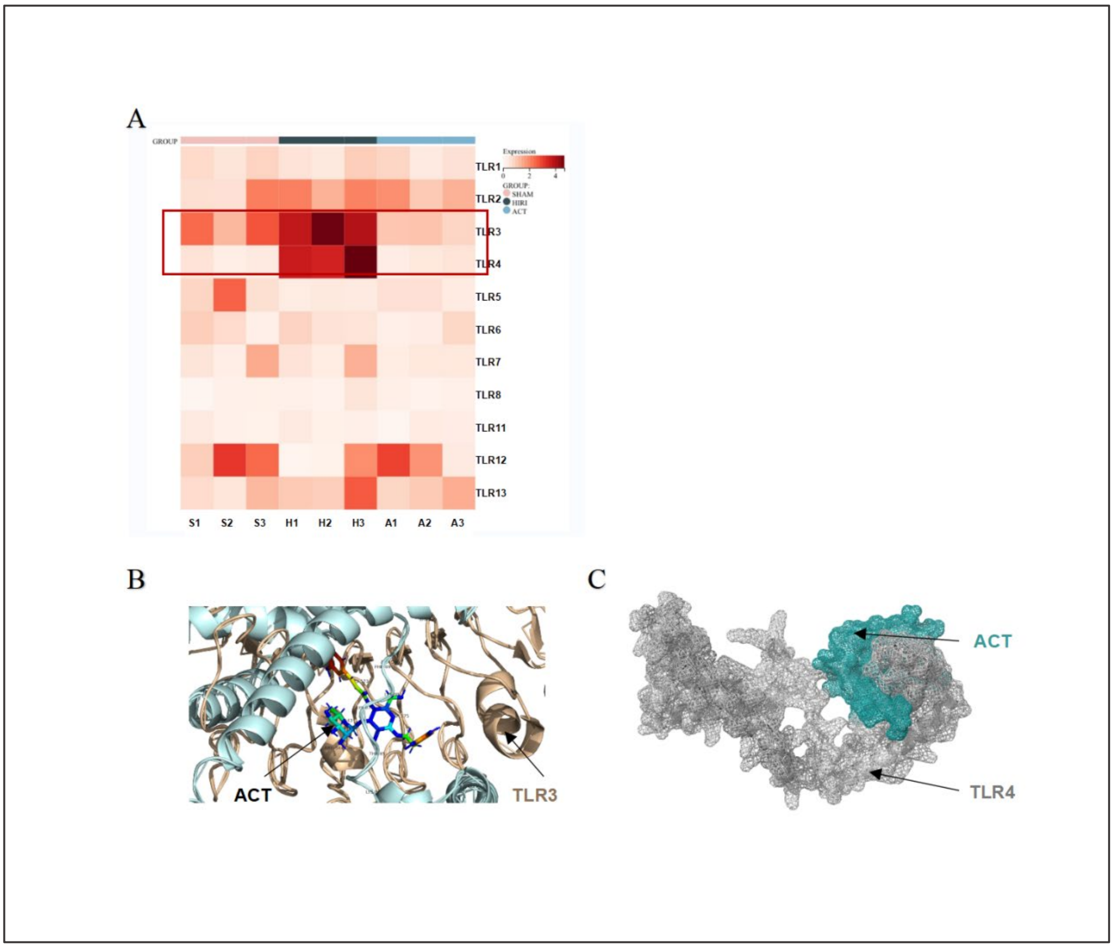

Fig. S6. The molecular docking sites of ACT and TLR3/4. (**A**) The expression mapping of TLRs in mRNA sequencing results of mouse liver. (**B**) The binding area simulation between ACT and TLR3. (**C**) The binding area simulation between ACT and TLR4.

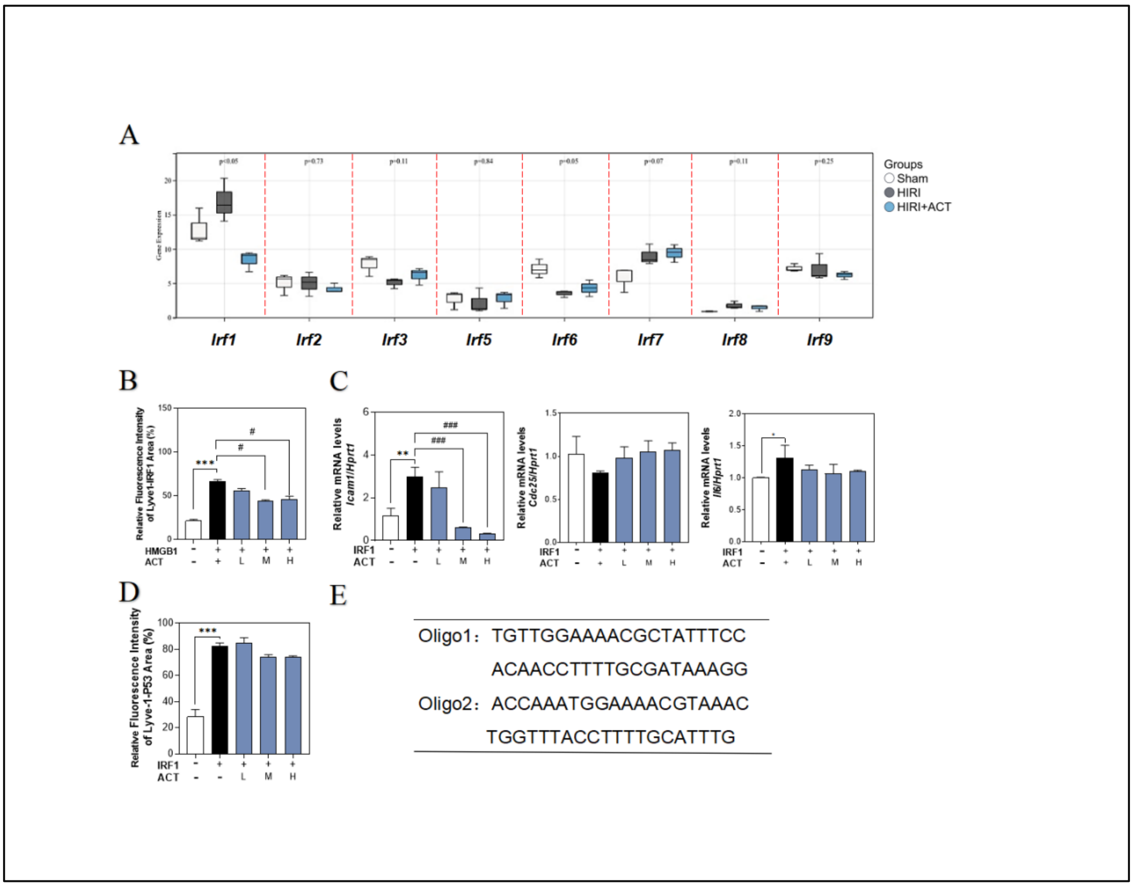

Fig. S7. The recombinant protein IRF1 facilitated the LSECs senescent process. (**A**) The gene expression of IRFs in mRNA-sequencing analysis results of primary LSECs. (**B**) The statistical charts of immunofluorescence staining of IRF1 and Lyve-1 of LSECs. (**C**) Relative expression of mRNA levels of *icam1*, *cdc25*, and *il6* of LSECs was measured by qPCR and further normalized with *hprt1*. (**D**) The predicted oligo sequence of transcription factor IRF1 and protein CXCL1. Statistical significance: $^{**}P < 0.01$; compared with the Con group; $^{\#}P < 0.05$, $^{\#\#}P < 0.01$, $^{\#\#\#}P < 0.01$ compared with the HMGB1 or IRF1 group.

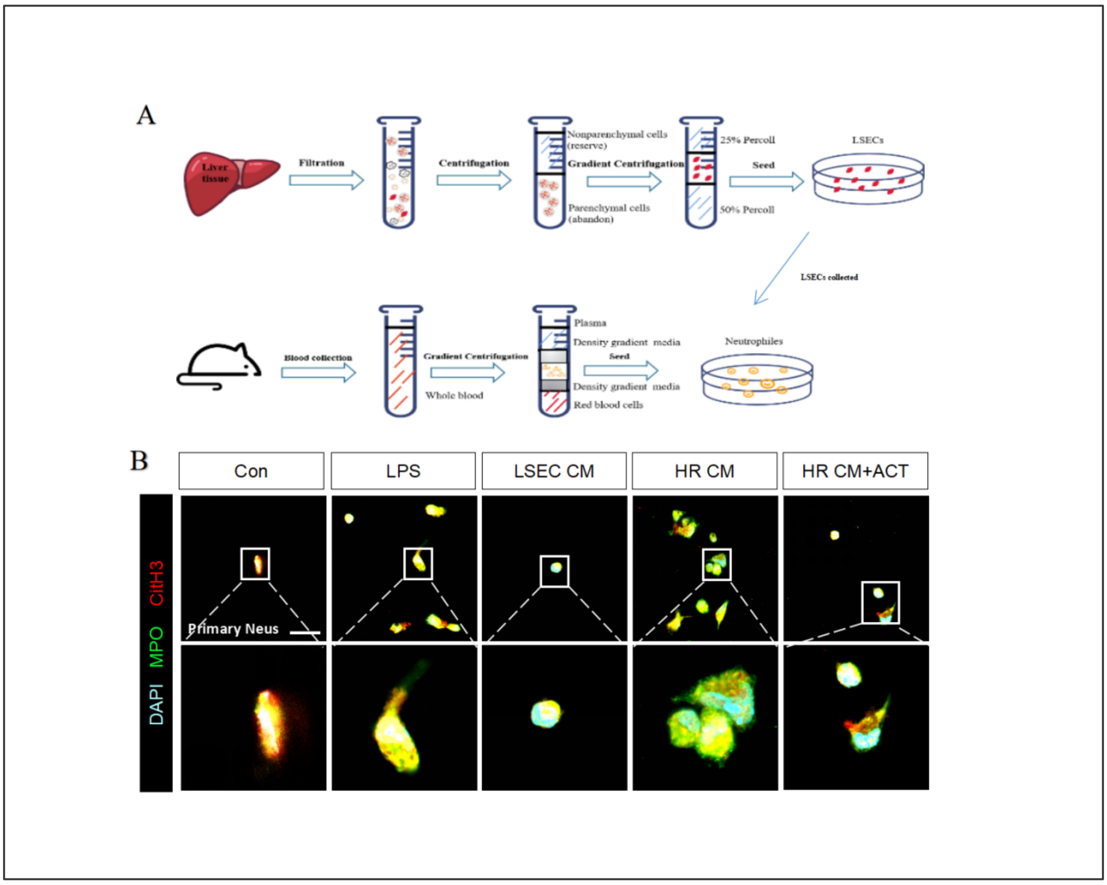

Fig. S8. The isolation and identification of primary neutrophils. (**A**) The flow chart of neutrophils isolation and identification. (**B**) The representative immunofluorescence images of citH3 and MPO for identification of neutrophils.

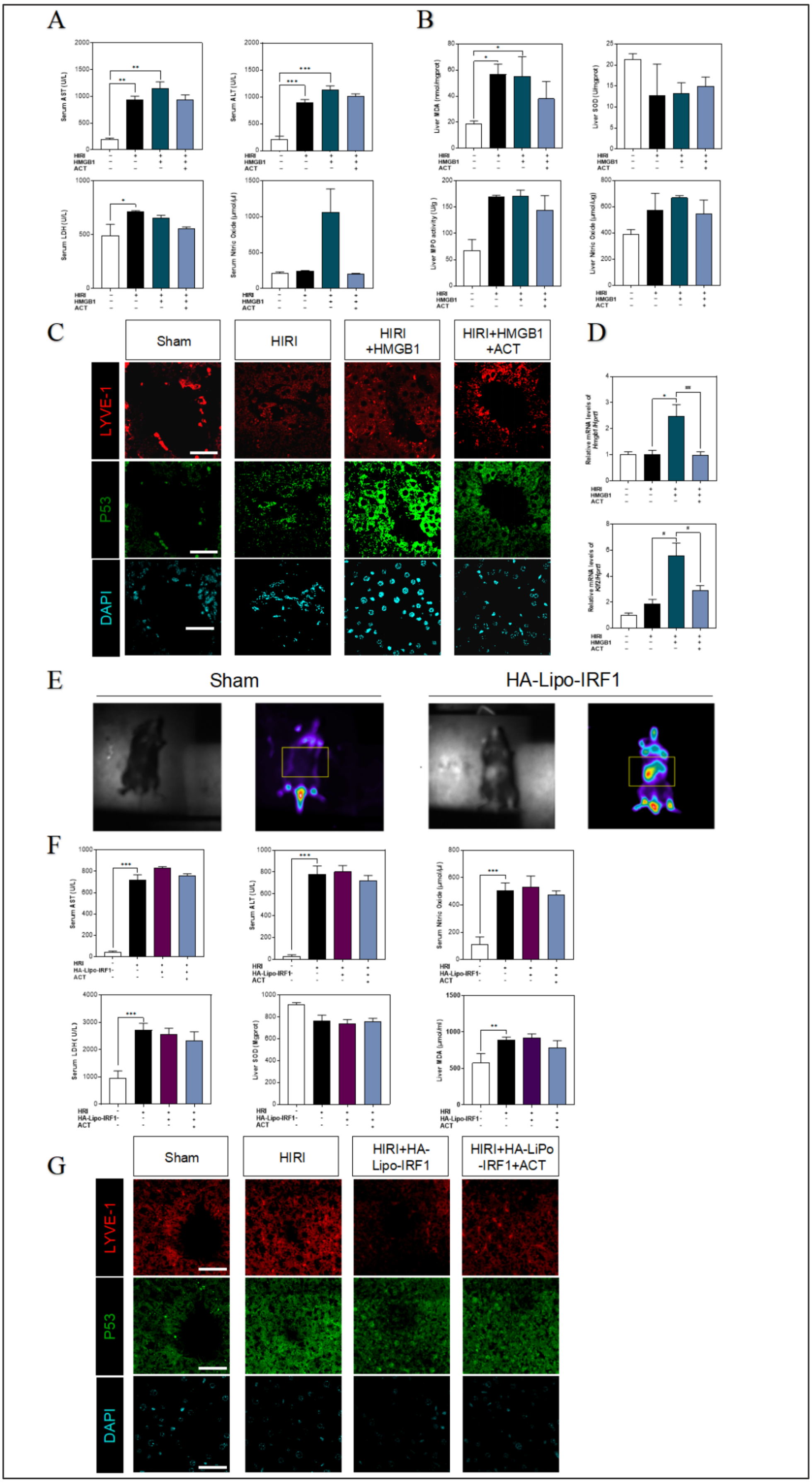

Fig. S9. The recombinant HMGB1 protein or overexpressed IRF1 liposome

blocks the hepatoprotective effects of ACT on HIRI. (**A**) Serum levels of AST, ALT, NO and LDH of mouse treated with recombinant HMGB1 protein and ACT. (**B**) The levels of MDA, SOD and NO in mouse liver under the recombinant HMGB1 protein and ACT treatment. (**C**) The fluorescent light channel in 594nm (LYVE-1), 488nm (P53) and DAPI of mouse liver treated with recombinant HMGB1 protein and ACT (scale bar = 40 μm). (**D**) The relative expression of mRNA levels of *hmgb1* and *klf2* in mouse liver treated with recombinant HMGB1 protein and ACT were measured by qPCR and further normalized with *hprt1*. (**E**) The representative immunofluorescence images of DIR-labeled HA-Lipo-IRF1 mouse. (**F**) The levels of AST, ALT, NO, and LDH in serum, and levels of SOD and MDA in the liver of mouse treated with HA-Lipo-IRF1 and ACT. (**G**) The fluorescent light channel in 594nm (LYVE-1), 488nm (P53), and DAPI of mouse liver treated with HA-Lipo-IRF1 and ACT (scale bar = 40 μm). Statistical significance: $^{*}P < 0.05$, $^{*}P < 0.01$, $^{***}P < 0.001$, compared with the sham group; $^{\#}P < 0.05$, $^{\#\#}P < 0.01$, $^{\#\#\#}P < 0.001$ compared with relative model groups (n = 6).

Supplementary material

Supplementary Table 1. The information of used antibodies.

Name	Abbreviation	Supplier	Cat no.
Albumin Monoclonal antibody	ALB	Proteintech	16475-1-AP
Lymphatic vessel endothelial hyaluronan receptor 1 Polyclonal antibody	LYVE-1	Proteintech	51011-1-AP
Cytokeratin 7 Polyclonal antibody	CK7	Proteintech	15539-1-AP
KI67 Polyclonal antibody	Ki67	Proteintech	27309-1-AP
Tumor protein P53 Polyclonal antibody	P53	Proteintech	60283-2-1g
High Mobility Group Protein 1 Polyclonal antibody	HMGB1	Proteintech	10829-1-AP
Interferon regulatory factor 1 Polyclonal antibody	IRF1	Proteintech	11335-1-AP
C-X-C motif chemokine ligand 1 Polyclonal antibody	CXCL1	Proteintech	12335-1-AP
Myeloperoxidase Polyclonal antibody	MPO	Proteintech	2225-1-AP
CD11c/Integrin Alpha X Polyclonal antibody	CD11	Proteintech	17342-1-AP
Anti-histone H3 (citrulline R2/R8/R17) antibody	CitH3	Abcam	ab5103
Hepatocyte nuclear factor 4 alpha antibody	HNF4a	Santa Cruz Biotechnology	61189
Alexa Fluor 594 anti-rabbit IgG secondary antibody	/	Cell Signaling Technology	8889S
Alexa Fluor 488 anti-mouse IgG secondary antibody	/	Thermo Fisher Scientific	A32723
